# Transcriptome of the Deep-Sea Black Scabbardfish, *Aphanopus carbo* (Perciformes: Trichiuridae): Tissue-Specific Expression Patterns and Candidate Genes Associated to Depth Adaptation

**DOI:** 10.1155/2014/267482

**Published:** 2014-09-17

**Authors:** Sergio Stefanni, Raul Bettencourt, Miguel Pinheiro, Gianluca De Moro, Lucia Bongiorni, Alberto Pallavicini

**Affiliations:** ^1^ISSIA-CNR, Via de Marini 6, 16149 Genova, Italy; ^2^LARSyS, Associated Laboratory & Centre of IMAR of the University of the Azores, Department of Oceanography and Fisheries, Rua Prof. Frederico Machado 4, 9901-862 Horta, Azores, Portugal; ^3^School of Medicine, University of St Andrews, North Haugh, St Andrews KY16 9TF, UK; ^4^Department of Life Sciences, University of Trieste, Piazzale Valmaura, 9, 34148 Trieste, Italy; ^5^Institute of Marine Sciences, National Research Council (ISMAR-CNR), Castello 2437/F, 30122 Venezia, Italy

## Abstract

Deep-sea fishes provide a unique opportunity to study the physiology and evolutionary adaptation to extreme environments. We carried out a high throughput sequencing analysis on a 454 GS-FLX titanium plate using unnormalized cDNA libraries from six tissues of *A. carbo*. Assemblage and annotations were performed by Newbler and InterPro/Pfam analyses, respectively. The assembly of 544,491 high quality reads provided 8,319 contigs, 55.6% of which retrieved blast hits against the NCBI nonredundant database or were annotated with ESTscan. Comparison of functional genes at both the protein sequences and protein stability levels, associated with adaptations to depth, revealed similarities between *A. carbo* and other bathypelagic fishes. A selection of putative genes was standardized to evaluate the correlation between number of contigs and their normalized expression, as determined by qPCR amplification. The screening of the libraries contributed to the identification of new EST simple-sequence repeats (SSRs) and to the design of primer pairs suitable for population genetic studies as well as for tagging and mapping of genes. The characterization of the deep-sea fish *A. carbo* first transcriptome is expected to provide abundant resources for genetic, evolutionary, and ecological studies of this species and the basis for further investigation of depth-related adaptation processes in fishes.

## 1. Introduction

The deep-sea (>1000 m depth) covers about 70% of the Earth's surface, representing one of the last large unexplored areas on the planet. Only within the last few decades the technology has advanced sufficiently to reach the deep-sea effectively, revealing unexpected high levels of biodiversity and extremely diverse habitats (canyons, cold seeps, hydrothermal vents, deep-water coral reefs, mud volcanoes, seamounts, and trenches) of significant conservation interest and potential high economic values. Deep-sea environments are characterized by extremely high hydrostatic pressures (1 MPa every 100 m), lack of light, and low temperatures (down to 1-2°C). Therefore, fish as well as any other organism living in the deep-sea had to adapt to tolerate conditions of this extreme habitat [1].

First studies on adaptation to high pressure and low temperatures are dated back in the ‘70s and they report comparison of common proteins present in shallow and deep-water fishes [2, 3]. Key enzymes in muscle tissues that exhibit adaptive differences among species at different depths are the lactate dehydrogenase (LDH) and malate dehydrogenase (MDH) presenting differences in structural stability (reviews in [4–6]). More recent studies on evolutionary adaptation of functional genes to high pressure report unique amino acid substitutions in *α*-skeletal actin and myosin heavy chain (MyHC) proteins in deep-sea fishes [7–10]. For deep-sea species inhabiting hydrothermal vents and cold seeps, environments characterized by high pressure, chronic hypoxia, and high concentrations of toxic compounds, molecular and functional adaptation of hemoglobins (Hbs) are reviewed in Hourdez and Weber [11]. Despite these studies, our knowledge on wide scale gene expression patterns in deep-sea fish remains elusive.

The black scabbardfish,* Aphanopus carbo* (Lowe 1839), is a bathypelagic species belonging to the Trichiuridae family and is distributed in temperate-cold Atlantic waters at depths between 200 and 1800 m [12, 13].* A. carbo* represents a commercially valuable species for several regions of the Iberic peninsula, especially in Madeira where catches have reached up to 1000 tons per year [14] amounting to ca. 55% of the total landings. Recently this species has become increasingly targeted by Portuguese, French, and Irish fishing fleets ([15] and literature therein) and fishery data have shown a constant decline in population [16]. The information available on the biology, maturity, spawning, and growth of this species [17, 18] is scattered. Recent studies are reporting a panmictic distribution of this species in the NE Atlantic with multiple breeding sites at low latitudes [19]. It is also worth mentioning that, in southern locations, this species lives in sympatry with* A. intermedius*, a close related species with very similar morphology [20], therefore attracting interest for evolutionary studies.

High-throughput sequencing approaches applied to transcriptomics now provide a global perspective on taxonomic and functional profiling of genes expectedly expressed under the influence of environmental conditions in which these organisms live. Also known as next-generation sequencing, these techniques allow for a massive characterization of expressed sequence tags (ESTs) providing an overview of those genes expressed in a given tissue at any given time [21]. In silico analyses of massive gene libraries may serve several interests among others. For instances, from discovery and identification of new genes, characterization of gene expression, to development of novel genetic markers for quantitative trait locus (QTL), and population of genomic analyses. The breadth of next-generation sequencing applications extends over a variety of biological questions including those addressing pertinent questions regarding a species' ecology, life history, and evolution [22, 23].

Previous studies regarding transcriptome sequencing and gene expression studies in deep-water species were mostly limited to hydrothermal vents invertebrates [24], microbial communities in hydrothermal plumes [25], deep-sediments [26], and in the water column [27] leaving vertebrates species virtually under-represented. The present work represents a pioneer study for deep-sea fishes providing new insights into the role of differential gene expression on the environmental adaptation of deep-sea black scabbardfish.

Here we describe the assembly and annotation of the transcriptome of* A. carbo* obtained by sequencing mRNA libraries of six tissues (spleen, brain, heart, gonads, liver, and muscle) and explore functional genes whose sequence might be associated to depth adaptation. Additionally, we tested the correlation of selected candidate genes comparing the number of contigs against the gene expression normalized to a relative value of 1.0, as determined by qPCR amplification. Furthermore, the screening of the libraries allowed the identification of new EST-simple-sequence repeats (SSRs) and the design of primer pairs suitable for population genetic studies as well as tagging and mapping of genes.

## 2. Methods

### 2.1. Fish and Tissue Samples

Specimens of* Aphanopus carbo* (two males and two females) were collected in 2009 onboard of the RV “*Arquipelago.*”* A. carbo* were fished at depth range 1100–1250 m using deep-water long-lines in proximity of the Condor Seamount, located approximately at 15 nm SW of the island of Fayal (Azores, Portugal). The four specimens used in this study were caught on the same longline set and once onboard, the freshly caught animals were dissected and portions (or complete organs) of spleen, brain, heart, gonads, liver, and muscle tissues were preserved in formamide solution and kept at −20°C until RNA extraction was performed.

To validate the correct identification of the species, a small portion of muscle tissue was also preserved in 95% ethanol for molecular screening following Stefanni et al. [28] protocols.

### 2.2. RNA Extraction and Sequencing

Total RNA was extracted from 20 to 40 mg of each of the six preserved tissues of a pool of four* A. carbo* individualsusing the RiboPure kit (Ambion, Applied Biosystems). Quantity and purity of the RNA was determined on a 1.4% agarose-MOPS-formaldehyde denaturing gels and by assessing the *A*_260/280_ and *A*_260/230_ ratios using the NanoVue spectrophotometer (GE Healthcare). Poly-A RNA was extracted from 15 *μ*g of each total RNA sample using the Poly(A)Purist mRNA Purification kit according to manufacturer's instructions (Ambion, Applied Biosystems). mRNAs were transcribed into cDNA utilizing Mint-2 cDNA synthesis kit (Evrogen, http://www.evrogen.com/) according to manufacturer's instructions for NGS platforms. Six cDNA libraries were constructed from mRNA of individual pools of tissues and sequenced in a single 454 GS FLX Titanium run. Each of the cDNA libraries was characterised by unique sequence tags (MIDs) that allowed to trace back the sequences generated from single tissues after assembly.

cDNAs were sheared by nebulization to yield random fragments approximately 500–800 bp in length, by applying 30 psi (2,1 bar) of nitrogen for 1 minute on 4 *μ*g of each library. The distribution of fragments was verified on a BioAnalyser DNA 7500 LabChip (Agilent Technologies). The fragmented cDNA sample was end-repaired with T4 DNA polymerase and T4 polynucleotide kinase and adaptor sequences ligated according to the manufacturer's instructions [29]. The fragments were immobilized onto streptavidin beads and nick-repaired with* Bst* polymerase. The cDNA fragments were denaturated with alkali to yield single stranded cDNA (sscDNA) library. Quality of the library was assayed on a BioAnalyser RNA 6000 Pico LabChip (Agilent Technologies) and quantity measured by spectrofluorimetry with the Quant-iT RiboGreen RNA Assay kit (Invitrogen). A titration was set up at 1, 2, 4, and 8 copies per bead (cpb) in the clonal amplification by emulsion PCR to optimize yield and sequence quality. The percent enrichment of beads carrying the sscDNA was determined and the amount of library input calculated to 18%. A large scale emulsion PCR was set-up based on the previous value and sequenced at Biocant (Cantanhede, Portugal) using the 454 GS FLX Titanium pyrosequencing on a full 70X75 PicoTiterPlate, according to manufacturer's instructions (Roche).

### 2.3. Bioinformatic Analyses

High quality reads were assembled using Newbler ver. 2.6 (Roche 454) sequence analysis software. All reads were identified and grouped by their unique MIDs to the tissue of origin. Trimming and masking the polyAs was a common procedure for the assembling tool.

The assemblage is characterized by read overlaps and multiple alignments made in nucleotide space. Consensus base-calling and quality value determination for contigs are performed in flow space. The use of flow space in determining the properties of the consensus sequence results in an improved accuracy for the final base-calls. The implementation of this software was performed using default parameters. Assembled contigs were annotated through sequence similarity searches against the National Centre for Biotechnology Information (NCBI) nonredundant (nr) protein database using the BLASTx [30] with a cut-off criterion of an expect-value (*e*-value) < 10^−6^. The contigs that did not find a hit were further processed with ESTScan (http://www.ch.embnet.org/software/ESTScan2.html). The two assemblages of amino acid sequences, resulting from the BLASTx searches at high level of stringency and the ESTScan, were processed by InterProScan for functional annotation of transcripts applying the function for the mapping of gene ontology (GO) terms. The GO method classifies genes within a hierarchy using a systematic nomenclature of attributes that can be assigned to all gene products independently from the organism of origin. To reduce the redundancy in the consensus sequences which correspond to the same gene we used BLASTClust to detect similar assemblies with 95% identity and 90% coverage. All the results from both assemblage methods were loaded into a SQL database developed for this purpose.

To validate the accuracy of the assembly, the resulting contigs were compared to previously sequenced transcriptomes of 6 teleosts including* Danio rerio*,* Gasterosteus aculeatus*,* Oreochromis niloticus*,* Oryzias latipes*,* Takifugu rubripes*, and* Tetraodon nigroviridis*, using tBLASTn [30] to find protein homologs at two levels of stringency (*e*-value < 10^−3^ and *e*-value < 10^−10^).

To identify protein conserved domain specific for each tissue analysed a new annotation was performed with Hmmer against the Pfam database (ver. 25.0). Protein domain representativeness for each tissue was obtained comparing protein domain abundance in a particular tissue versus all the tissues compiled together using a hypergeometric test.

### 2.4. cDNA Synthesis and qPCR Validation Tests

Fresh cDNA was synthesised from the six mRNAs that were used for pyrosequencing, cDNA synthesis was performed using primers with oligo(dT) and the ThermoScript RT-PCR System (Invitrogen) following the manufacturer's instructions.

A set of 28 genes were selected including candidates that were tissue-specific and genes that were encountered in the tissue expressed at similar as well as at different amounts in all the six libraries, with the aim of covering most of the possible expression scenarios within the dataset. Frequencies of contigs for all candidates genes in the mRNA libraries were obtained by detecting orthologous gene sequences using the BLAST tool included in the* A. carbo* database.

For the design of all qPCR primers (Table 1) we used the web interface NCBI Primer-BLAST (http://blast.ncbi.nlm.nih.gov/). Alignments of the sequences provided by the output from the internal blast search were used to select all primer sets.

Gene expression, calculated as relative expression, was determined by means of real-time PCR using the CFX96 (Bio-Rad). Primer concentrations and sample dilutions were optimized to meet highest efficiency in the PCR reaction in a total reaction volume of 20 *μ*L. Fluorescent signal was determined by the addition of SsoFast EvaGreen Supermix (Bio-Rad) which was included in the cocktail accordingly to manufacturer's instructions. Baseline and threshold cycle were always set to automatic in the sequence detection software, CFX Manager (Bio-Rad). All plates contained a “no template control” (NTC) and each sample was tested in duplicate. Cycling conditions for gene amplifications were 95°C for 3 min followed by 35 cycles of 95°C for 10 s, 56°C for 10 s, and 68°C for 15 s. An additional protocol for melting curves analysis included a cycle at 95°C followed by a progressive reading of fluorescence for every cycle from 65°C to 95°C for 5 s at intervals of 0.5°C. Gene expression, normalized to a relative value of 1.0 for all the genes selected and for each tissue, was compared to the contigs frequencies generated by the assembly platform to determine the significance of correlation between qPCRs values and 454 sequencing reads from unnormalized cDNA libraries.

### 2.5. Characterization of Depth-Related Functional Genes

The predicted amino acid sequences of functional genes of* A. carbo *possibly related to depth adaptations were compared to those deposited in NCBI database searching for homologies. We aligned and compared translated sequences of lactate dehydrogenase (LDH-A and LDH-B), cytosolic malate dehydrogenase (MDHc), hemoglobins (Hb-A and Hb-B), actin (ACTA1), and myosin heavy chain (MyHC). Protein and nucleotide sequences were aligned using Clustal X [31] while sequence analysis and phylogenetic inferences were performed using CLC Main Workbench (v. 6.8.2, CLC Bio). The neighbor-joining (NJ) algorithm [32] was implemented to construct a phylogenetic tree using HKY substitution model and attributing a gap penalty of 10. The support for internal branches was assessed using the bootstrap [33] with 1000 replicates.

Nucleotide alignments and ML trees built, implementing the most appropriate substitution model under the Akaike Information Criterion (AIC), were used in the program “codeml” in PAML 4 [34] to assess selective pressure on those genes for which the complete sequences was available. Positive (or negative) selected sites were defined by the ratio between nonsynonymous* versus* synonymous substitutions (*dN*/*dS* or *ω*). Two models were tested comparatively: M1 which groups codons in two classes (*ω* < 1 and *ω* = 1), clustering sites under negative or neutral selection; and M2 which groups codons in three classes (*ω* < 1, *ω* = 1 and *ω* > 1), adding a cluster for sites under positive selection to the ones defined in M1. Probabilistic measures of how well these models fits the evolutionary relationship of individual genes were calculated from the likelihood values of fitted models and the number of “free parameters” for all genes.

Protein stability was estimated using a virtual quantification software [35] calculating Gibbs free energy in terms of kinetic and thermodynamic quantities taking into account each amino acid contribution for the maintenance of the native structure of the protein. For a protein to maintain its stability there is a need of sufficient hydrophobic residues which will utilize free energy to guide proper folding [35].

### 2.6. EST-SSR Resources for Population Genetics

Among various molecular markers, simple sequence repeats (SSRs) are highly polymorphic, easier to develop, and very useful for researches such as genetic diversity assessment. Therefore* A. carbo* database was further used to detect such regions in the transcriptome sequencing data and provide a list of combination of primer sets on flanking regions that could be used for population genetic studies. To identify EST-SSRs, all the contigs were searched using MISA [36] and for primer design we used Primer3 [37]. The algorithm of the SSR finder identifies a good quality repeat when one locus is present with adjacent loci at an up or downstream distance higher than 100 bp and parameters were set to locate a minimum of 20 bp sequence repeats: di-mers (x12), 3-mers (x8), 4-mers (x5), 5-mers (x5), and 6-mers (x5). Primer design was performed setting parameters of a minimum size of 20 bp and melting temperatures of 60°C.

## 3. Results and Discussion

### 3.1. Sequences Assemblage and Functional Annotations

**Figure 1:**
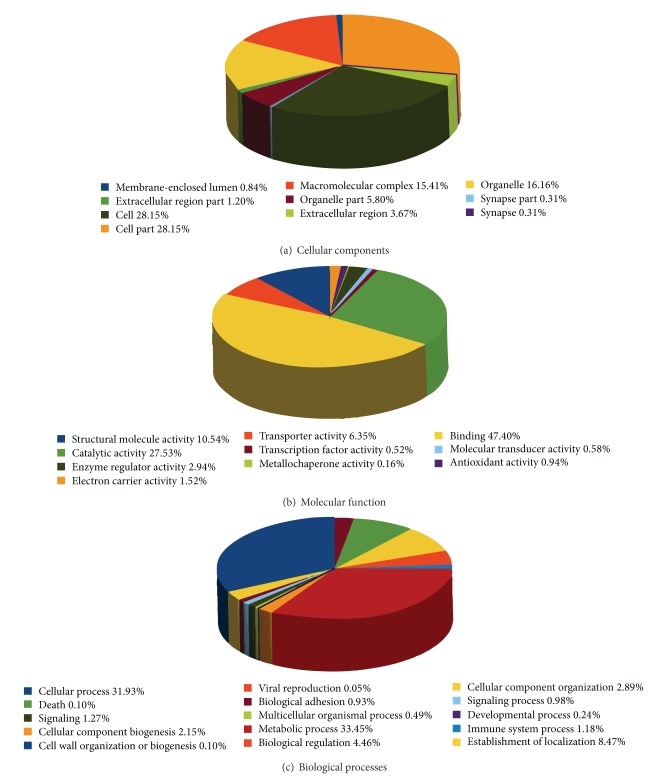
Functional categorization of unigenes with gene ontology (GO) term for the* Aphanopus carbo* EST collection. These unigenes results were functionally classified from the six tissues pooled together as percentages under three main functional categories with respective GO Slim terms. Data refer to assemblage derived by implementation of Newbler, Roche 454 sequence analysis software.

After sequence trimming, a total of 544,491 high quality reads were produced with an average length of 237 bp corresponding to 129.5 Mb. A total of 8,319 contigs were assembled with Newbler (ver 2.6) as high quality consensus sequences without the presence of singletons. A summary of EST data for each of the six tissues is reported in Table 2. A total of 2,440 assembled contigs were annotated against the NCBI nonredundant protein database at the cut-off (*e*-value) < 10^−6^. Additional 3,843 contigs with no protein matches were further processed with ESTScan to find 2,715 homologous proteins. All the contigs were then processed by InterProScan for functional annotation of transcripts and mapping functional information to gene ontology (GO) terms. Of the 5,155 amino-acid sequences (out of the 6,283 totally sequenced), 1,728 could be annotated within the GO hierarchy (Figure 1), while 2,395 could be annotated accordingly to InterProScan. The complete GO mapping of* A. carbo* for individual tissues can be accessed on the dedicated online database (http://transcriptomics.biocant.pt/AphanopusCarbo/). The largest proportions of GO functional categories are of similar proportion to the unnormalized libraries of Tilapia [38]. In the Cellular Components GO group (Figure 1(a)), the genes involved in cell and cell part functional categories corresponded to the largest percentage of the pie chart (28.15% each). In the Molecular Function GO group (Figure 1(b)), almost half of the total genes were involved in binding (47.40%), followed by those related to catalytic activity (27.53%). Finally, for the Biological Processes GO group (Figure 1(c)), the largest portion of the genes were involved in metabolic processes (33.45%), tightly followed by the category of genes linked to the Cellular Process (31.93%).

To further validate the accuracy of the A. carbo assembly, we compared our dataset to the transcriptomes of six other prior sequenced teleosts including* Danio rerio*,* Gasterosteus aculeatus*,* Oreochromis niloticus*,* Oryzias latipes*,* Takifugu rubripes*, and* Tetraodon nigroviridis*. Similarity searches were performed comparing our assembled contigs against each of the available transcriptomes using tBLASTn [30]. The results highlighted strong similarity between the transcriptomes of* A. carbo* and other teleosts indicating that about 30% of the total contigs matched protein homologs (*e*-values <10^−3^ ranging between 2,268 and 2,457) and that our assembly presents the highest similarity (by little margin) with the transcriptome of Gasterosteus aculeatus (*e*-value < 10^−10^ = 2,223) (Table 3).

A total of 1639 (20%) transcripts were annotated by Pfam protein domains matches and the set of protein domains characterizing each single tissue was identified by hypergeometric test (Table 4).

### 3.2. qPCR Assays and Validation Tests

**Figure 2:**
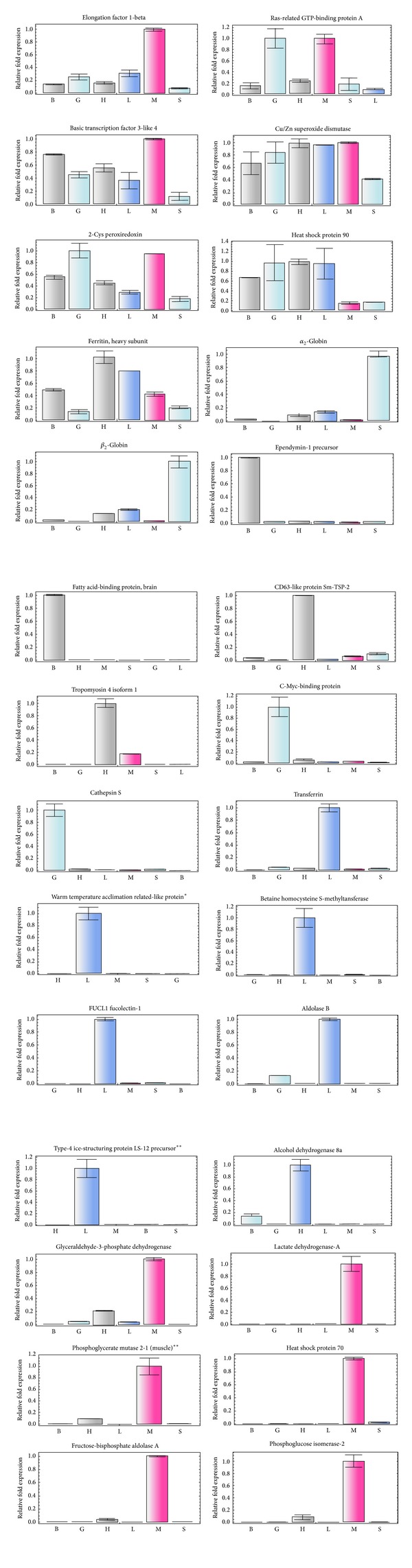
Gene expression normalized to a relative value of 1.0 of all genes selected for this study in different tissues from* Aphanopus carbo*. S = spleen, B = brain, H = heart, G = gonad, L = liver, M = muscle,  *= expression in the brain was below detection and  **= expression in the gonads was below detection.

Quantitative PCR assays were performed using cDNA samples at 1 : 400 dilution (up to 100 ng approximately in the qPCR master mix), conditions at which the PCR resulted to be more efficient. We tested seven series of dilutions ranging from 1 : 50 to 1 : 1000. Based on optimized qPCR conditions; all targeted cDNAs were tested across all tissues for the complete set of genes selection used in this study. Normalized expression to a relative value of 1.0 for 28 genes is shown in Figure 2 (see: Figure S1 in Supplementary Material available online at http://dx.doi.org/10.1155/2014/267482).

**Figure 3:**
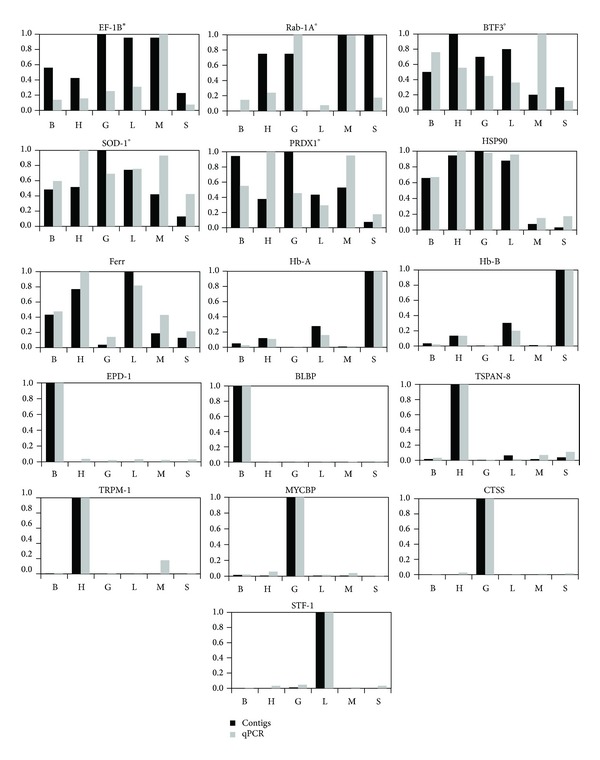
Comparative plots of relative expression as contig frequencies* versus* mean qPCR values for all genes selected for this study. Codes for each of the gene are listed in Table 1, S = spleen, B = brain, H = heart, G = gonad, L = liver, and M = muscle; the correlation coefficient (Pearson's *r*) resulted to be highly significant (*P* < 0.0001) for most of the genes except for *P < 0.05 and °not significant.

Statistical tests were performed to identify the correlation between the mean value of normalized expression and a relative value of 1.0, as determined by qPCR against contig frequencies using Pearson's *r* coefficient and its significance (Figure 3). Most of the genes showed highly significant correlation; however one case indicated a reduced significance (elongation factor) and a few other cases resulted to be not significant (e.g., Ras-related GTP-binding protein, Basic Transcription Factor 3, Cu/Zn Superoxide Dismutase and 2-Cys Peroxiredoxin). Reduced or lack of significance appeared to be related to a low number of contigs from the 454 sequencing (Table 5), which might be regarded as an indication of failure to reach library saturation. Low values in contig frequency should correspond to a reduced level of expression as the libraries are nonnormalized; therefore caution should be used when exploring 454 outputs below a certain threshold. Our data derived from the reading of a single plate were insufficient for systematical comparison of all the genes, although the majority of the genes show a high correlation between the 454 data and the qPCR. However, it should be emphasized that this exercise was not meant to be a surrogate to the qPCR approach for the study of gene expression but more a proxy for a preliminary screening of differentially expressed genes in multiple libraries.

### 3.3. Candidate Genes Associated to Depth

**Figure 4:**
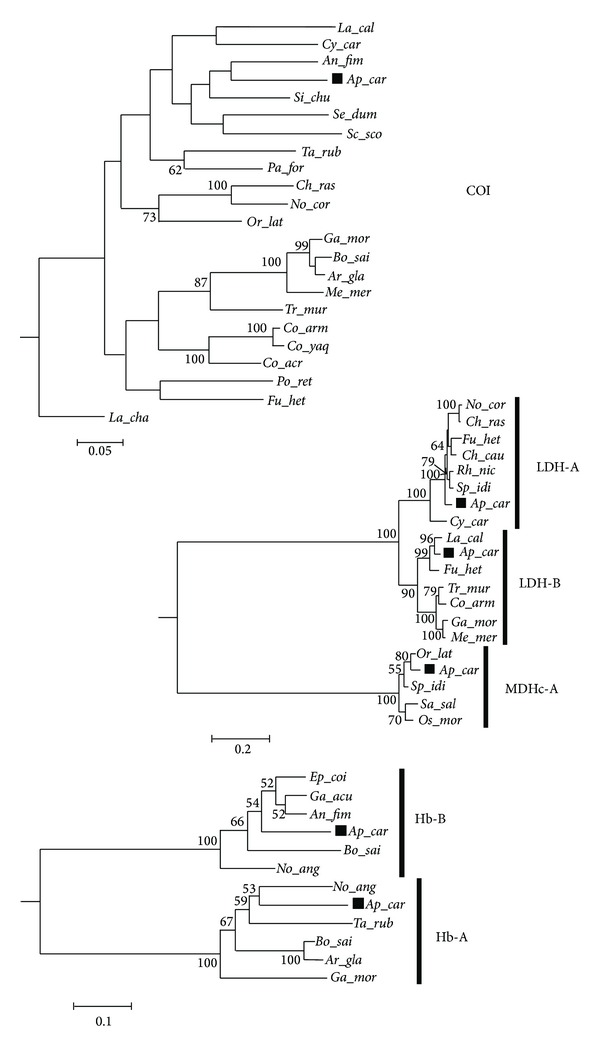
At the top: molecular phylogenetic tree of COI gene estimated by the HKY nucleotide substitution model constructed by neighbor joining and rooted including the sequence of* Latimeria chalumnae* (acc. nr.: NC_001804); in the middle: NJ tree of LDH and MDH genes; and at the bottom: NJ tree of globin (Hb) gene. Numbers in proximity of the nodes denote the bootstrap value (above 50%) out of 1000 replicates. The scale indicates the evolutionary distance of the base substitution per site. Taxon coding includes the first two letters for the genus followed by three letters for the species name (see Table 6). A black square helps to locate* Aphanopus carbo *in the trees.

The predicted amino acid sequences of* A. carbo* functional genes putatively related to depth adaptations were compared to those deposited in NCBI database for homologies searches. Complete alignments of orthologous protein sequences from the set of functional genes that have been reported as responsive to depth adaptations included representatives of Teleosts belonging to several families (Table 6). Exploring the levels of similarities expressed as percentage of amino acid identity or number of amino acid that differ among sequences between* A. carbo* and other fish species (Table 7) brings evidence supporting the fact that deep-living as well as polar species are not the most similar to the black scabbard fish. We attempted to reconstruct the phylogenies for those species using amino acid sequences of functional genes (LDH-A, LDH-B, MDHc-A, Hb-A and Hb-B) and corresponding mtCOI nucleotide sequences (Figure 4). The resulting trees showed similar topologies, suggesting that the signals embedded in these functional genes reflect evolutionary divergence among taxa rather than any enzyme adaptations relationships.

The lactate dehydrogenase A (LDH-A: isotig01401) was encountered abundantly in the muscle tissue (Table 5, Figure 2) and its comparison with other orthologous sequences differs for a number of amino acids between 16 and 44. One indel at position 75 had to be added to the two polar species to obtain a complete alignment (Table 7). The percentage of identity was higher with the strictly marine species ranging between 95.2 and 93.7%. Unfortunately, no sequences from neither deep-sea nor abyssal fishes were available for comparison. On the other hand, lactate dehydrogenase B (LDH-B: isotig01423) in* A. carbo* was highly represented in the heart while very scarcely recorded in the other tissues (Table S1). The largest sequence divergence in terms of amino acid substitutions ranges between 47 and 55 when compared with deep-water macrourids, and similarly to LDH-A, an indel had to be added in the sequences of the gadiform species at position 76 to obtain a complete alignment (Table 7). Previous studies on benthopelagic fish report a significant decline of LDH activities with increasing water depth [39]. However, it is known that variation in enzyme activities of fish at a given depth is influenced by feeding behaviour and locomotory modes [40, 41].

Cytosolic malate dehygrogenase A (MDHc-A: isotig01010) was detected in most of the tissues, with the exception of the muscle (Table S1). An isoform of MDHc was also detected but its sequence covered only the last 110 amino acids (isotig01731). However the type of substitutions and tissue expression pattern (Table S1) according to Merrit and Quattro [42], lead us to believe that this cytosolic isoform is MDHc-B. It has been reported that the two teleost MDHc isozymes are the products of a gene duplication event after the separation of teleosts and tetrapods (see [42] and references therein) although the exact timing of this duplication has not been inferred.

Further comparison analyses were carried out only for MDHc-A, with orthologous sequences obtained from NCBI database where only shallow-water fish sequences were available. The complete alignment did not include any indel, and sequences differed from* A. carbo* between 15 and 35 amino acid substitutions, representing a percentage of identity ranging from 95.5 to 89.5%.

Two *α*-skeletal actins were detected from the* A. carbo* database: the isoform 1 (isotig01459), expressed in shallow-water species and probably not functioning in abyssal fishes, and the isoform 2a (isotig01561). The mutations specific for* A. carbo* in the actin 2a were the substitutions Asp3Glu, Ala155Ser (a mutation also common with the isoform 2b [7]), Ser234Val, Val165Ile, Leu261Val, and finally Ala278Thr. This type of isoform was reported in other deep-sea species as* Coryphaenoides acrolepis*,* C. cinereus*,* C. yaquinae,* and* C. armarus* [7]. The isoform 2b, so far reported only in abyssal species, was not detected in* A. carbo*.

Actins 1 and 2a were significantly expressed in muscle tissue with evidence of its presence also in the heart (Table S1). Direct comparison of* A. carbo* actin 1 with orthologous sequences reported for other marine and deep-water species differed by just 1 amino acid at position 3 (Table 7). The number of substitutions increases to 2 when this sequence is compared to the isoform 2a of* Coryphaenoides *species (substitution at position 155) and to 5 amino acids replacement (Table 7) when compared to the isoform 2b, two of which are unique (positions 116 and 137) [7]. Actin has a function in polymerization of G-actin to F-actin in neutral salts. While the volume is increased following polymerization, the reaction is strongly affected by high pressure [43]. It is reported that the substitutions of Val54Ala or Leu67Pro reduced the volume change associated with actin polymerization [7].

The assemblage of the myosin heavy chain protein (MyHC: isotig02568 and isotig1394) obtained in* A. carbo* was incomplete (All_gs454_000756 and All_gs454_000001) containing a gap of 711 amino acids at the positions from 171 to 882 of the 1933 AA of the complete sequence. Unfortunately, within this gap there are the two loop regions with characteristic structures that are uniquely reported for deep-sea fish: loop-2 region is shorter and the loop-1 region has a proresidue [9]. MyHC was almost uniquely found in muscle tissue (Table S1) and for comparison analyses with orthologous genes from other fish we only used the last 305 AA (All_gs454_000184) of the complete sequence. The lowest number of amino acid substitutions ranged between 53 and 69 (corresponding to sequence identity between 94.95 to 93.43%) when the sequence from* A. carbo *is compared to its relative shallow water marine and freshwater species, while this value increases up to 92 amino acid substitutions (and corresponding sequence identity of 91.25%) when compared to its relatives from deep water or polar regions (Table 7).

Variation in globin sequences was analysed exploring the *α*_2_- and *β*_2_-chains (Hb-A: isotig00247 and Hb-B: isotig00163), whose relative expressions were detected more abundantly in the spleen, followed by liver, heart, brain, and muscle and virtually absent in the gonads (Figure 2, Table 5). Comparison analyses with orthologous sequences of deep-sea gadiforms and a notothenoid indicate that the number of amino acid substitutions ranged between 39 and 53 (Hb-A) and between 31 and 42 (Hb-B). The complete alignments included the additional single indel for the *α*_2_-chain of* Notothenia angustata* at position 102. Notothenioids acquired a completely different globin genotype with respect to other teleostean groups. The Antarctic ichthyofauna (dominated by a single taxonomically uniform group) lost its globin multiplicity in correlation with temperature stability. On the other hand, for the Arctic ichthyofauna it may have been advantageous to maintain a multiple globin system, helping to deal with environmental changes and metabolic demands [44].

Selective pressure in the site-by-site patterns among species was evaluated for LDH-A, -B, MDHc, and ACTA1 (isoform 1). There was no evidence of positive selection at the nucleotide site level in any of those genes whose global *ω* value was very low in all cases (from 0 in ACTA1 and 0.032 in LDH-B) (Table S2). The proportion of sites supporting positive selection, the model M2, was null (LDH-A and -B) or extremely low (0.3% in MDHc and 1.6% in ACTA1) (Table S2). These results indicate that the evolution of these four genes in teleosts is constrained by very stringent selective pressure (model probabilities for M1* versus* M2 ranging from 87% in MDHc and 88% in all others).

**Figure 5:**
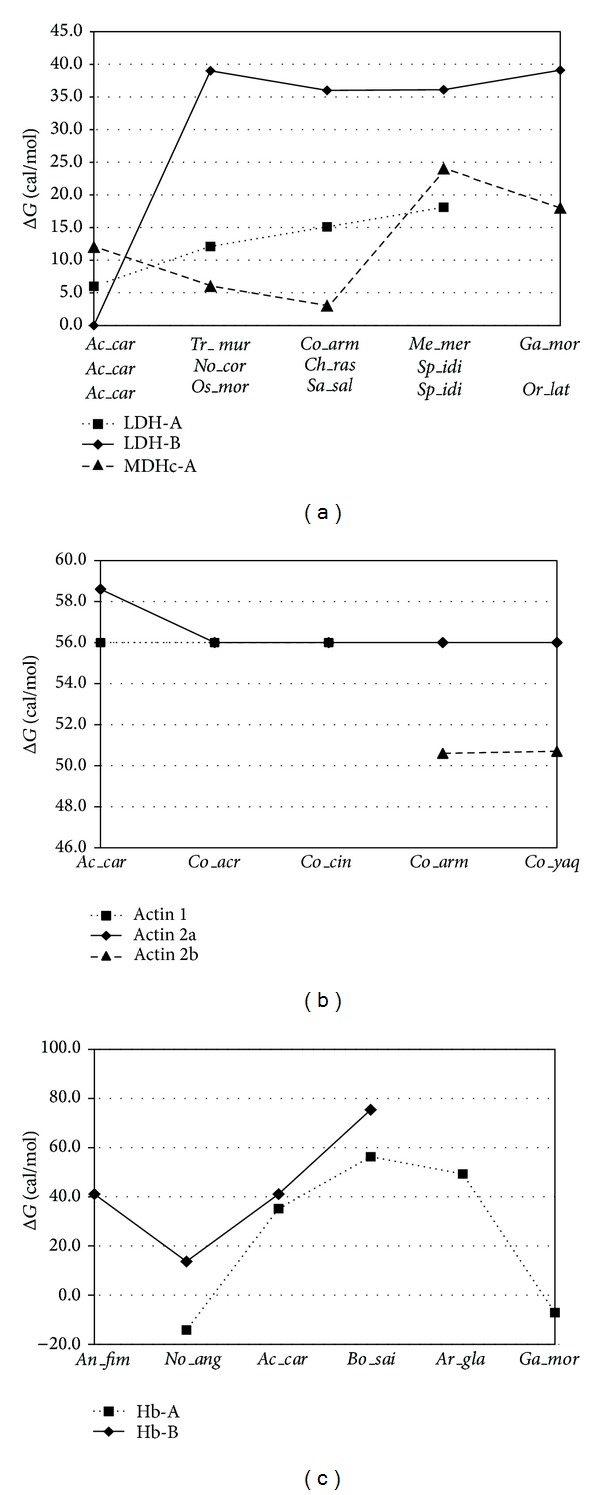
Comparative plots of normalised difference in protein stability (Δ*G*) for different functional genes among several representative species. Taxon coding includes the first two letters for the genus followed by three letters for the species name (see Table 6).

In terms of protein stability calculated as Gibbs free energy and taking into consideration kinetic and thermodynamic quantities, we explored only the functional genes for which the complete sequences were obtained (Figure 5). In this context, protein stability is defined by the ability of a protein to retain its structural conformation or its activity when subjected to physical or chemical manipulations; therefore the energy consumed by activation to promoted folding has to be compensated by thermal stability provided by the energy of denaturation [35]. Hence, protein stability is quantitatively calculated by the standard Gibbs energy change (Δ*G*), allowing comparison of stabilities for different proteins [45].

The normalised difference in protein stability (Δ*G*) of lactate dehydrogenase A (LDH-A) was lower in* A. carbo* compared to fish from polar waters and tropical marine (Figure 5). This gradual variation was primarily dictated by the lower contributions of leucine and lysine in the hydrophobic trend of kinetic calculation in the* A. carbo *sequence (Table S3). The substantial drop in Δ*G* of LDH-B in* A. carbo* compared to the orthologous sequences of polar, abyssal, and shallower marine fishes (Figure 5) was due to a larger contribution of glutamic acid (Table S3) promoting the thermal denaturation of the protein. Other studies investigating kinetic, physical properties and ability to withstand high pressure of LDH-B of two gadiformes support our calculations showing that the enzyme from the deep-sea species has a significant increased tolerance to pressure and higher thermostability [6]. To provide protection to* A. carbo* LDH-B from pressure and temperature denaturation, osmolytes might play an essential role. Experiments adding Trimethylamine-N-oxide (TMAO) to samples resulted in substantial increment of activity of LDH-B under conditions at which the enzyme was previously sensitive [6].

In the cytosolic malate dehygrogenase A (MDHc-A) there was a sharp decrease in protein stability of marine fishes going from shallower to deeper waters (no sequences of abyssal species were available). Lowest values are encountered in the sequences of the two euryhaline species included in the comparison (Figure 5). This progressive decrement in stability was associated to the larger contribution of aspartic acid (Table S3), also a promoter of thermal denaturation of the protein. The responses to pressure and temperature of soluble enzymes like LDH and MDH differ adaptively among species found at different depths.

The isoforms 1 and 2a of *α*-skeletal actin of* A. carbo* showed very similar numerical values of Δ*G* compared to their homologues of deep-water (actin 1) and deep and abyssal water (actin 2a). Protein stability drops substantially when these two isoforms were compared with actin 2b, only present in abyssal species (Figure 5). Such reduction in stability was due to a higher number of hydrophobic amino acids contributing to the thermodynamic calculation (Table S3). Morita [7] reported that the substitutions of Gln137Lys and Ala155Ser generate a mechanism for stabilizing enzyme-substrate interactions under high pressure.

Comparing the stability of globin, *α*_2_-chain (Hb-A), in shallow, deep-water, and polar fish, the larger Δ*G* was found in polar and deep-water cods, followed by* A. carbo* before dropping to negative values for the shallower representatives of Antarctic and temperate environments (Figure 5). Stability of protein in* A. carbo* and its deep-water relatives was linked to larger contributions by leucine in the kinetic calculations promoting folding and thermal stability (Table S3). The stability as Δ*G* of globin sequences of the *β*_2_-chains (Hb-B) showed a very similar trend as in Hb-A (although there were no negative values), and the two deep-living species had very similar protein stability value. Variation in Δ*G* among environments was linked to the balanced contribution of hydrophobic amino acids promoting folding* versus* those lowering thermal stability (Table S3).

Although for key enzymes it was found that adaptive differences among species at different depths showed structural changes as well as structural stability (reviews in: [4–6]), several studies remarked on the importance of regulatory regions of the genome acting on gene regulation (see [46]). Promoter regions often work together with other* cys*-regulatory elements (e.g., transcription factors binding sites, enhancers, silencers, and insulators) to regulate the transcription and expression of mRNA in a specific tissue at different times and places throughout the genome. In addition, in regards to adaptation to depth, it is remarked the importance of osmolyte concentrations of methylamines (TMAO is the most relevant). These are protein stabilizers that counteract inhibition of proteins by hydrostatic pressure [47–49].

Finally, we should also take into account the stress imposed on this fishes being captured at a depth range of 1100–1250 m and brought to surface in at least 3–5 hours. By the time specimens of black scabbardfish reach the surface they are already dead or dying; therefore the expression of some of the transcripts might be different if compared to a transcriptome of a fish sampled at a thousand meters depth. Notwithstanding, several experiments using model species have been targeting specific genes linked to stress factors [50] and this might prove useful in interpreting transcriptomes of animals undergoing stressful conditions before being sampled.

On the bases of this preliminary but encouraging results, we are planning to explore further deep water adaptivity employing different NGS sequence technology and experimental design, for instance using individual RADseq approach on a larger number of specimens caught at deep or shallow waters or by comparing sister species that live at different depth levels.

### 3.4. EST-SSR Resources for Population Genetics

In this study, a total of 153 EST-SSRs were detected in 142 contigs (3.87%) with a frequency of one EST-SSR per 27,69 kb sequence (Table 8). A further selection was made taking into account the type of repeats, size of the fragment, and quality of the primer sets, reducing the EST-SSR to 98. Some of those microsatellite markers were found in one tissue but not in others (16.3%), while others resolved to be found in more than one tissue (83.7%). Among the identified EST-SSRs, tri-nucleotide repeats represented the largest portion (40.8%), followed by di-nucleotide (28.6%), and tetra-nucleotide (25.7%), and only 2 EST-SSRs penta-nucleotide were identified (Table S4). Primer pairs have sizes ranging between 19 and 25 bp and melting temperatures from 57.1°C to 61.2°C, while expected PCR products are 100 to 280 bp in length. None of these EST-derived primer pairs have been tested for neutrality; therefore not all may be suitable for basic population genetic studies.

## 4. Conclusions

The transcriptome analysis of* A. carbo*, revealed a comprehensive set of genes expressed in six tissues, producing over 8,000 genes, 55.6% of which annotated by similarity to known proteins or nucleotide sequences from freely accessible database. The transcriptome of black scabbardfish sets the stage for expanding the genetic resources available while providing sets of genes that are likely tied with physiological adaptations to depth, particularly to low temperature and high pressure factors. This study represents the first transcriptome analysis for a deep-sea fish providing insights based on comparison analyses of homologous depth-related functional genes from shallow, deep-water, and abyssal fish highlighting similarities for* A. carbo* isozyme patterns and stability to other bathypelagic fishes. Direct sequence comparison suggested that the signals embedded in these functional genes reflect evolutionary divergence among taxa rather than any kind of enzyme adaptations. Organisms are adapted to deep-sea environment and their physiological tolerance may vary among taxa as well as their enzymatic activities under extreme conditions. Osmolyte concentrations as protein stabilizers that counteract inhibition of proteins by hydrostatic pressure [6, 47–49] play a key role in deep-sea adaptation. Furthermore, contribution to adaptation is also provided by promoter regions that together with* cys*- and* trans*-regulatory elements work concertedly, at the mRNA transcription level, to drive the expression of a gene in a specific tissue or at a specific time [46].

The strong correlation detected between the values of standardized contigs frequency with the expression level of the gene by qPCR supports the use of our data to explore gene expression patterns in* A. carbo*.

Finally, considering the importance of this species for fishery management, a further exploration of this database has provided the characterization of new EST-SSR markers as additional resource for basic population genetic studies as well as tagging and mapping of genes.

## Supplementary Material

As supplementary material we included: amplification plots of all genes selected for this study in different tissue from *Aphanopus carbo* (Figure S1); a summary table of contig frequency in all tissues for those genes associated to depth not listed in Table 3 (Table S1); details on outputs related to the selection models for four teleost genes associated to depth (Table S2); values of Gibbs free energy calculations in terms of kinetic and thermodynamic quantities for all depth-related genes (Table S3); and the list of EST-SSR markers (Table S4).

## Figures and Tables

**Table 1:** List of targeted genes using qPCR, primer sets specifically designed for this study, size for each of the product, and NCBI accession number for all the EST sequences.

Gene	Primer name	Primer Sequences (5′-3′)	Size (bp)	NCBI Accession #
Elongation factor 1-beta	*EF-1B L *	GCTTGGACATGTCGGTCTCGTC	229 bp	All_gs454_000396
	*EF-1B H *	GTGGCTGACACCACATCTGGC		
Ras-related GTP-binding protein A	*Rab-1A L *	AGTAGCCGTTCCACCTTGTCGG	247 bp	All_gs454_000598
	*Rab-1A H *	TGCCAAGAAACCGTACGTGGGA		
Basic Transcription Factor 3-like 4	*BTF3 L *	CCCAAAGTTCAGGCCTCCCTGT	273 bp	All_gs454_000873
	*BTF3 H *	TCATGTGCGTCAGTTCGCTTCG		
Cu/Zn Superoxide Dismutase	*SOD-1 L *	AAACGTGACTGCAGGAGGGGAT	240 bp	All_gs454_000925
	*SOD-1 H *	CAGTGCTCCTGCTCCATGTTCG		
2-Cys Peroxiredoxin	*PRDX1 L *	CCGATAACCTCGCAGCCGATAC	243 bp	All_gs454_000558
	*PRDX1 H *	ACAGTCATTTGCCACCAGCATCA		
Heat Shock Protein 90	*HSP90 L *	TGACGATGTCCCCACAGATGAGG	221 bp	All_gs454_000008
	*HSP90 H *	GCAACACTGGTCCACCACACAAC		
Ferritin, heavy subunit	*Ferr L *	CCTGCAGCTTGAGAAGAGCGTC	203 bp	All_gs454_000681
	*Ferr H *	CAAACAGGTACTCGGCCATGCC		
*α*_2_ Globin	*Hb-A L *	AAATTGTTGGCCATGCGGAGGA	208 bp	All_gs454_001919
	*Hb-A H *	CTGAGGTTCAGCAGACCTGCCT		
*β*_2_ Globin	*Hb-B L *	TCGTCTACCCCTGGTGTCAGAG	245 bp	All_gs454_001018
	*Hb-B H *	AACCACAATGGTCAGGCAGTCC		
Ependymin-1 precursor	*EPD-1 L *	CAGGTGTGAGGCAGTGCAGT	230 bp	All_gs454_000469
	*EPD-1 H *	ACCCCGATCTCCTCCTGGTG		
Fatty acid-binding protein, brain	*BLBP L *	CAACACTTCTTGGCCGGTTTGG	239 bp	All_gs454_001220
	*BLBP H *	GAGAGGAGTTCGACGAAGCCAC		
CD63-like protein Sm-TSP-2	*TSPAN-8 L *	TCGCTGGCTGCTCTGAGAAAGA	200 bp	All_gs454_000381
	*TSPAN-8 H *	GGTCACGCCGAGCTGTATTCTG		
Tropomyosin 4 isoform 1	*TRPM-1 L *	GTGGAGGAGGAGTTGGACCGAG	221 bp	All_gs454_000222
	*TRPM1 H *	TTGCGAGCCACCTCCTCGTATT		
C-Myc-binding protein	*MYCBP L *	CGCCAGTTTACCTGCGTTCCAA	182 bp	All_gs454_001640
	*MYCBP H *	GGCCGTCAACAACACCACCTTT		
Cathepsin S	*CTSS L *	AACAGCCTACCCCTACACAGCC	200 bp	All_gs454_000156
	*CTSS H *	TGTACACACCGTGGCGGTAGAA		
Transferrin	*STF-1 L *	AGCTGCACCAGCTTCACAGTTG	215 bp	All_gs454_000004
	*STF-1 H *	AAGGATGGCACCAGACAACCCA		
Warm Temperature Acclimation related-like 65 kDa protein	*HPX L *	TGATACCGGGTGGAACCTGGTG	207 bp	All_gs454_000060
	*HPX H *	GCTGCTGTGGAGTGTCCCAAAG		
Betaine Homocysteine S-methyltransferase	*BHTM L *	GGGGGTTCGCTGTTACCAAGTG	194 bp	All_gs454_000088
	*BHTM H *	TGTGAGACAGCAGCCTCAGGAG		
FUCL1 Fucolectin-1	*FUCL1 L *	CGCAAACCCTTTGGCTGGTGTA	196 bp	All_gs454_000758
	*FUCL1 H *	GGCTTTTCCTTGGACTGCCAGG		
Aldolase B	*ALDB L *	GCCATTGGTCTTGGCCCTGATC	220 bp	All_gs454_000115
	*ALDB H *	CGCTGTGCCTGGTATCTGCTTC		
Type-4 ice-structuring protein LS-12 precursor	*ISP LS12 L *	AAGACCTGACAAACCAGGCCCA	198 bp	All_gs454_001277
	*ISP LS12 H *	GGAGGATGGCCTCCATCTGCTT		
Alcohol Dehydrogenase 8a	*ADH L *	GGCAAGAAGGTGCTGCAGTTCA	228 bp	All_gs454_000105-6
	*ADH H *	CATGACTGCAGCCAAACCCACA		
Glyceraldehyde-3-phosphate Dehydrogenase	*GAPDH L *	GTCAACCACTGACACGTTGGGG	229 bp	All_gs454_000148
	*GAPDH H *	CGGCATCATTGAGGGCCTGATG		
Lactate Dehydrogenase-A	*LDH-A L *	TCTTAACCTGGTGCAGCGCAAC	219 bp	All_gs454_000149
	*LDH-A H *	TGGAGCTTCTCGCCCATGATGT		
Phosphoglycerate Mutase 2-1 (muscle)	*PglyM L *	ACACCTCTGTGCTGAAACGTGC	212 bp	All_gs454_000309
	*PglyM H *	CATGGGTGGAGGTGGGATGTCA		
Heat Shock Protein 70	*HSP70 L *	CGGTGTTGTGTGCTGGGTGAAA	207 bp	All_gs454_000005
	*HSP70 H *	CCACATAGCTGGGTGTGGTCCT		
Fructose-bisphosphate Aldolase A	*FBPA L *	GGAACCAACGGCGAGACAACAA	208 bp	All_gs454_002732
	*FBPA H *	CAATGGGGACGATGCCATGCAT		
Phosphoglucose Isomerase-2	*PGI L *	CCACACTGGGCCAATTGTCTGG	217 bp	All_gs454_000011
	*PGI H *	GGCCTCCTCTGTGGTCTTACCC		

**Table 2:** Global statistics for each of the nonnormalized libraries using Newbler software.

Tissue	Spleen	Brain	Heart	Gonad	Liver	Muscle	Total
Total EST	15,034	33,337	73,263	157,275	134,523	92,788	544,491
Total bases	3,426,510	8,219,500	17,647,600	37,123,700	31,792,600	23,342,900	129,412,000
Contigs	567	651	1,260	3,875	1,274	626	8,319
Average contig length	470	619	567	465	612	689	555
Contigs *e*-value < 10^−6^	220	420	622	951	617	409	2,440
ESTscan	584	345	977	2,838	634	269	2,715
No similarity	36	70	109	406	211	74	1,128
GO annotation	202	338	473	623	509	307	1,728
InterPro annotation	223	417	610	908	649	395	2,395

**Table 3:** Similarity search for unigenes comparing transcriptomes of other teleosts against *Aphanopus carbo* using two levels of stringency.

Species	Sequences available	*A. carbo* *e*-value < 10^−3^	*A. carbo* *e*-value < 10^−10^
*Danio rerio *	42,787	2,457	2,199
*Gasterosteus aculeatus *	27,576	2,445	2,223
*Oreochromis niloticus *	26,763	2,436	2,202
*Oryzias latipes *	24,674	2,412	2,173
*Takifugu rubripes *	47,841	2,268	2,062
*Tetraodon nigroviridis *	23,118	2,300	2,073

**(a):** 

Gonads				Heart				Liver			
Pfam ID	Domain	Freq	*P*	Pfam ID	Domain	Freq	*P*	Pfam ID	Domain	Freq	*P*
PF00100	Zona pellucida	17	3.15 10^−13^	PF00011	HSP20	3	4.03 10^−4^	PF00084	Sushi	14	1.44 10^−10^
PF00125	Histone	8	6.06 10^−5^	PF13405	EF hand 4	5	6.77 10^−4^	PF00089	Trypsin	20	7.59 10^−9^
PF01400	Astacin	3	3.22 10^−3^	PF00412	LIM	3	1.52 10^−3^	PF00079	Serpin	12	5.63 10^−6^
PF00069	Pkinase	3	0.01	PF05556	Calsarcin	3	3.60 10^−3^	PF07678	A2M comp	4	1.35 10^−4^
PF00653	BIR	2	0.02	PF01576	Myosin tail 1	3	3.60 10^−3^	PF01042	Ribonuc L-PSP	3	1.26 10^−3^
PF13424	TPR 12	2	0.02	PF00056	Ldh 1 N	3	3.60 10^−3^	PF00045	Hemopexin	3	1.26 10^−3^
PF13695	zf-3CxxC	2	0.02	PF05300	DUF737	2	5.50 10^−3^	PF00059	Lectin C	6	1.69 10^−3^
PF09360	zf-CDGSH	2	0.02	PF00992	Troponin	4	6.40 10^−3^	PF00701	DHDPS	4	4.64 10^−3^
PF01712	dNK	2	0.02	PF00595	PDZ	3	6.82 10^−3^	PF00386	C1q	8	6.63 10^−3^
PF00538	Linker histone	2	0.02	PF00022	Actin	3	0.01	PF00021	UPAR LY6	5	0.01
PF10178	DUF2372	2	0.02	PF02874	ATP-synt ab N	2	0.02	PF00754	F5 F8 type C	9	0.01
PF04856	Securin	2	0.02	PF13895	Ig 2	3	0.02	PF08702	Fib alpha	2	0.01
PF00250	Fork head	2	0.02	PF00191	Annexin	2	0.03	PF03982	DAGAT	2	0.01
PF01498	HTH Tnp Tc3 2	3	0.03	PF00212	ANP	2	0.03	PF01048	PNP UDP 1	2	0.01
PF00268	Ribonuc red sm	2	0.06	PF05347	Complex1 LYR	2	0.07	PF01014	Uricase	2	0.01

**(b):** 

Muscle				Brain				Spleen			
Pfam ID	Domain	Freq	*P*	Pfam ID	Domain	Freq	*P*	Pfam ID	Domain	Freq	*P*
PF01410	Troponin	7	1.99 10^−5^	PF01669	Myelin MBP	4	4.03 10^−5^	PF00042	Globin	5	1.33 10^−6^
PF02807	COLFI	3	9.67 10^−4^	PF01453	B lectin	2	6.44 10^−3^	PF00078	RVT 1	5	1.33 10^−6^
PF00041	ATP-gua PtransN	3	3.59 10^−3^	PF00612	IQ	2	6.44 10^−3^	PF00993	MHC II alpha	4	5.02 10^−6^
PF02453	fn3	3	3.59 10^−3^	PF11414	Suppressor APC	2	6.44 10^−3^	PF07686	V set	5	2.49 10^−6^
PF13895	Reticulon	3	3.59 10^−3^	PF05768	DUF836	2	6.44 10^−3^	PF07654	C1 set	5	4.71 10^−4^
PF00365	Ig_2	4	7.97 10^−3^	PF05196	PTN MK N	2	6.44 10^−3^	PF00089	Trypsin	5	2.01 10^−3^
PF01216	PFK	2	9.84 10^−3^	PF04300	FBA	2	6.44 10^−3^	PF01391	Collagen	2	2.30 10^−3^
PF01267	Calsequestrin	2	9.84 10^−3^	PF00287	Na K-ATPase	2	6.44 10^−3^	PF00240	ubiquitin	3	3.28 10^−3^
PF00261	F-actin cap A	2	9.84 10^−3^	PF11032	ApoM	3	8.53 10^−3^	PF09307	MHC2-interact	2	6.67 10^−3^
PF00856	Tropomyosin	2	9.84 10^−3^	PF00061	Lipocalin	4	0.01	PF00643	Zf-B box	2	0.01
PF01661	SET	2	9.84 10^−3^	PF00007	Cys knot	2	0.02	PF13445	Zf-RING LisH	2	0.01
PF01667	Macro	2	0.03	PF01275	Myelin PLP	2	0.02	PF01498	HTH Tnp Tc3 2	2	0.02
PF00036	Ribosomal S27e	2	0.05	PF00300	His Phos 1	2	0.02	PF02301	HORMA	1	0.05
PF01576	efhand	2	0.05	PF00230	MIP	2	0.02	PF14259	RRM 6	1	0.05
PF01410	Myosin_tail_1	2	0.08	PF00091	Tubulin	3	0.02	PF09004	DUF1891	1	0.05

**Table 5:** Summary table of contig frequency for each of the tissues for those genes used to test the correlation with qPCR assays. S: spleen, B: brain, H: heart, G: gonad, L: liver and M: muscle. Names of genes based on their coding used in this table are found in table.

Gene	Contig code	Spleen	Brain	Gonads	Heart	Liver	Muscle
EF-1B	isotig00991	9	24	33	19	38	54
Rab-1A	isotig02689	4	0	3	3	0	4
BTF3	isotig02213	3	5	7	10	8	2
SOD-1	isotig02222	4	16	32	16	25	14
PRDX1	isotig01838	4	50	59	17	23	27
HSP90	isotig01406	3	64	94	86	86	13
Ferr	isotig01665	33	121	10	210	264	47
Hb-A	isotig06973	406	17	2	24	108	1
Hb-B	isotig00163	2,303	81	16	313	707	22
EPD-1	isotig01567	0	1,190	0	1	0	0
BLBP	isotig02632	0	171	0	0	0	0
TSPAN-8	isotig01397	17	7	3	407	24	9
TRPM-1	isotig01595	1	4	0	637	2	2
MYCBP	isotig01988	0	2	135	1	1	1
CTSS	isotig01659	0	0	135	0	0	0
STF-1	isotig00767	0	1	30	0	3,218	0
HPX	isotig01479	0	0	0	0	1,234	0
BHTM	isotig01489	1	0	0	0	1,024	0
FUCL1	isotig00473	1	0	0	0	22	0
ALDB	isotig01524	0	1	30	0	369	0
ISP LS12	isotig03004	0	0	0	0	211	1
ADH	isotig01491-546	1	2	16	7	292	6
GAPDH	isotig00609	0	8	51	539	134	1,281
LDH-A	isotig01401	2	7	2	5	0	789
PglyM	isotig01642	0	0	0	36	0	241
HSP70	isotig01398	0	0	0	0	2	142
FBPA	isotig00492	2	2	0	233	0	4,471
PGI	isotig01410	0	0	0	26	0	151

**Table 6:** Species list with informations on their type of environment (M = marine, BR = brackish and FW = freshwater), climate, depth range (in meters) and the type of genes used in the study with relative Genbank accession numbers.

Order	Family	Species	Common name	Environment	Climate	Depth range	Gene	NCBI Acc. Nr
Perciformes	Trichiuridae	*Aphanopus carbo *	Black scabbardfish	M	Deep-water	200–1700	COI	EU854076
Beloniformes	Adrianichthydae	*Oryzias latipes *	Japanese rice fish	FW + BR	Subtropical	shallow	ACTA1, MDHc, MyHC, COI	NM_001104806, NM_001163134, XM_004071618, AB498066
Scorpaeniformes	Hexagrammidae	*Pleurogrammus azonus *	Okhotsk atka mackerel	M	Temperate	0–240	ACTA1	AB073381
Perciformes	Scombridae	*Scomber scombrus *	Atlantic mackerl	M	Temperate	0–200 (0–1000)	ACTA1, COI	EF607093, KC015895
Perciformes	Percihcthyidae	*Siniperca chuatsi *	Mandarin fish	FW	Temperate	10	ACTA1, MyHC, COI	AY395872, AY454304, NC_015822
Perciformes	Sparidae	*Sparus aurata *	Gilthead seabream	M	Temperate	1–30 (1–150)	ACTA1	AF190473
Perciformes	Sphyraenidae	*Sphyraena idiastes *	Pelican barracuda	M	Tropical	3–24	ACTA1, LDH-A, MDHc, mMDH	AF503593, SIU80001, AF390559, AF390561
Tetraodontiformes	Tetraodontidae	*Takifugu rubripes *	Japanese pufferfish	M + FW + BR	Temperate	0–200 (0–1000)	Hb-A, mMDH, COI	XM_003964767, XM_003965959, HM102315
Scorpaeniformes	Anoplopomatidae	*Anoplopoma fimbria *	Sablefish	M	Deep-water	0–2740	Hb-B, COI	BT082849, JQ353978
Perciformes	Serranidae	*Epinephelus coioides *	Orange-spotted grouper	M + BR	Subtropical	1–100	Hb-B	GU982530
Gasterosteiformes	Gasterosteidae	*Gasterosteus aculeatus *	Three-spined stickleback	M + FW + BR	Temperate	0–100	Hb-B	NM_001267638
Perciformes	Nototheniidae	*Notothenia coriiceps *	Black rockcod	M	Polar	0–550	LDH-A, MyHC, COI	AF079822, AJ243767, EU326390
Perciformes	Nototheniidae	*Notothenia angustata *	Maori chief	M	Temperate	0–100	Hb-A, Hb-B	P62363, P29628
Gadiformes	Macrouridae	*Coryphaenoides armatus *	Abyssal grenadier	M	Deep-water	282–5180	LDH-B, MyHC, COI	AJ609232, AB330140, FJ164497
Gadiformes	Gadidae	*Gadus morhua *	Atlantic cod	M + BR	Temperate	0–600	LDH-B, COI	AJ609233, KC015385
Gadiformes	Gadidae	*Arctogadus glacialis *	Arctic cod	M	Deep-water	0–1000	Hb-A, COI	Q1AGS4, KC015200
Perciformes	Latidae	*Lates calcarifer *	Barramundi	M + FW + BR	Tropical	10–40	LDH-B, COI	FJ439507, JQ431879
Gadiformes	Gadidae	*Merlangius merlangus *	Whiting	M	Temperate	30–100 (10–200)	LDH-B, COI	AJ609234, JQ623954
Cyprinodontiformes	Poeciliidae	*Poecilia reticulata *	Guppy	FW + BR	Tropical	Shallow	LDH-B, COI	EF408825, JX968696
Gadiformes	Macrouridae	*Trachyrincus murrayi *	Roughnose grenadier	M	Deep-water	0–1630	LDH-B, COI	AJ609235, AP008990
Perciformes	Channichthyidae	*Chionodraco rastrospinosus *	Ocellated icefish	M	Polar	0–1000	LDH-A, COI	AF079829, EU326337
Perciformes	Pomacentridae	*Chromis caudalis *	Blue-axil chromis	M	Tropical	15–55	LDH-A	AY289558
Perciformes	Gobiidae	*Rhinogobiops nicholsii *	Blackeye goby	M	Subtropical	?-106	LDH-A	AF079534
Cypriniformes	Cyprinidae	*Cyprinus carpio *	Common carp	FW + BR	Subtropical	shallow	LDH-A, MyHC, COI	AF076528, D89992, HQ960709
Cyprinodontiformes	Fundulidae	*Fundulus heteroclitus *	Mummichog	M + FW + BR	Temperate	shallow	LDH-A, LDH-B, COI	L43525, L23792, EU524629
Osmeriformes	Osmeridae	*Osmerus mordax *	Rainbow smelt	M + FW + BR	Temperate	0–425	MDHc, mMDH	BT075651, BT075600
Salmoniformes	Salmonidae	*Salmo salar *	Atlantic salmon	M + FW + BR	Temperate	10–23 (0–210)	MDHc, mMDH	BT060183, BT048216
Gadiformes	Macrouridae	*Coryphaenoides acrolepis *	Pacific grenadier	M	Deep-water	900–1300	MyHC, COI	AB330141, JQ354060
Gadiformes	Macrouridae	*Coryphaenoides yaquinae *	n.a.	M	Deep-water	3400–5800	MyHC, COI	AB330139, GU440291
Perciformes	Cirrhitidae	*Paracirrhites forsteri *	Blackside hawkfish	M	Tropical	5–35	MyHC, COI	AJ243770, HQ561521
Perciformes	Carangidae	*Seriola dumerili *	Greater amberjack	M	Subtropical	18–72 (1–360)	MyHC, COI	AB032020, KC015917
Gadiformes	Gadidae	*Boreogadus saida *	Polar cod	M + BR	Polar	0–400	Hb-A, Hb-B, COI	DQ125471, Q1AGS6, KC015250

**Table:** (a) LDH-A (332 AA)

All_gs454_00149* vs. *	Diff.	Id. %	Gaps	Acc. nr.
*Sphyraena idiastes *	16	95.18	0	U80001
*Rhinogobiops nicholsii *	17	94.88	0	AF079534
*Chromis caudalis *	21	93.67	0	AY289558
*Chionodraco rastrospinosus *	26	92.17	1	AF079829
*Notothenia coriiceps *	27	91.87	1	AF079822
*Fundulus heteroclitus *	27	91.87	0	L43525
*Cyprinus carpio *	44	86.75	0	AF076528

**Table:** (b) MDHc-A (333 AA)

All_gs454_00146* vs. *	Diff.	Id. %	Gaps	Acc. nr.
*Oryzias latipes *	15	95.50	0	NM_1163134
*Sphyraena idiastes *	20	93.99	0	AF390559
*Osmerus mordax *	30	90.99	0	BT075651
*Salmo salar *	35	89.49	0	BT060183

**Table:** (c) Actin-1 (375 AA)

All_gs454_00104* vs. *	Diff.	Id. %	Gaps	Acc. nr.
*Scomber scombrus 1 *	0	100	0	EF607093
*Iniperca chuatsi 1 *	0	100	0	AY395872
*Oryzias latipes 1 *	1	99.73	0	NM_1104806
*Pleurogrammus azonus 1 *	1	99.73	0	AB073381
*Coryphaenoides acrolepis 1 *	1	99.73	0	AB021649
*Coryphaenoides cinereus 1 *	1	99.73	0	AB021651
*Coryphaenoides armatus 2a *	2	99.47	0	AB086240
*Coryphaenoides yaquinae 2a *	2	99.47	0	AB086242
*Coryphaenoides acrolepis 2a *	2	99.47	0	AB021650
*Coryphaenoides cinereus 2a *	2	99.47	0	AB021652
*Cyprinus carpio 1 *	2	99.47	0	AY395870
*Sphyraena idiastes 1 *	3	99.20	0	AF503593
*Coryphaenoides armatus 2b *	5	98.67	0	AB086241
*Coryphaenoides yaquinae 2b *	5	98.67	0	AB086243
*Aphanopus carbo 2a *	6	98.40	0	All_gs454_00102
*Sparus aurata 1 *	9	97.60	0	AF190473

**Table:** (d) LDH-B (334 AA)

All_gs454_00143* vs. *	Diff.	Id. %	Gaps	Acc. nr.
*Lates calcarifer *	14	95.81	0	FJ439507
*Fundulus heteroclitus *	21	93.71	0	L23792
*Trachyrincus murrayi *	47	85.93	0	AJ609235
*Coryphaenoides armatus *	50	85.03	0	AJ609232
*Merlangius merlangus *	52	84.43	1	AJ609234
*Gadus morhua *	55	83.53	1	AJ609233

**Table:** (e) MyHC^1^ (305 AA)

All_gs454_00001* vs. *	Diff.	Id. %	Gaps	Acc. nr.
*Paracirrhites forsteri *	53	94.95	0	AJ243770
*Seriola dumerilii *	54	94.86	0	AB032020
*Siniperca chuatsi *	59	94.38	0	AY454304
*Oryzias latipes *	62	94.10	2	XM_4071618
*Cyprinus carpio *	69	93.43	2	D89992
*Coryphaenoides acrolepis *	86	91.82	1	AB330141
*Notothenia coriiceps *	86	91.82	1	AJ243767
*Coryphaenoides yaquinae *	89	91.53	1	AB330139
*Coryphaenoides armatus *	92	91.25	1	AB330140

**Table:** (f) Hb-A^1^ (143 AA)

All_gs454_01074* vs. *	Diff.	Id. %	Gaps	Acc. nr.
*Notothenia angustata *	39	72.73	1	P62363^2^
*Boreogadus saida *	43	69.93	0	DQ125471
*Arctogadus glacialis *	44	69.93	0	DQ125475
*Takifugu rubripes *	46	67.83	0	XM_3964767
*Gadus morhua *	53	62.94	0	O42425^2^

**Table:** (g) Hb-B^1^ (146 AA)

All_gs454_01018* vs. *	Diff.	Id. %	Gaps	Acc. nr.
*Epinephelus coioides *	27	81.63	0	GU982530
*Anoplopoma fimbria *	31	78.91	0	BT082849
*Gasterosteus aculeatus *	31	78.91	0	NM_1267638
*Boreogadus saida *	40	72.79	0	Q1AGS6^2^
*Notothenia angustata *	42	71.43	0	P29628^2^

**Table 8:** Statistics of EST-SSRs identified in *Aphanopus carbo* transcriptome.

Searching item	Numbers
Total number of sequences examined	7,920
Total size of examined sequences (bp)	4,235,839
Total number of identified SSRs	153
Number of SSR containing sequences	142
Number of sequences containing more than 1 SSR	9
Number of SSRs present in compound formation	8
Di-nucleotide	63
Tri-nucleotide	52
Tetra-nucleotide	35
Penta-nucleotide	3

## References

[1] Somero GN (1992). Adaptations to high hydrostatic pressure. *Annual Review of Physiology*.

[2] Childress JJ, Nygaard MH (1973). The chemical composition of midwater fishes as a function of depth of occurence off southern California. *Deep-Sea Research and Oceanographic Abstracts*.

[3] Childress JJ, Somero GN (1979). Depth-related enzymic activities in muscle, brain and heart of deep-living pelagic marine teleosts. *Marine Biology*.

[4] Somero GN (1992). Biochemical ecology of deep-sea animals. *Experientia*.

[5] Sebert P (2002). Fish at high pressure: a hundred year history. *Comparative Biochemistry and Physiology A*.

[6] Brindley AA, Pickersgill RW, Partridge JC, Dunstan DJ, Hunt DM, Warren MJ (2008). Enzyme sequence and its relationship to hyperbaric stability of artificial and natural fish lactate dehydrogenases. *PLoS ONE*.

[7] Morita T (2003). Structure-based analysis of high pressure adaptation of α-actin. *Journal of Biological Chemistry*.

[8] Morita T (2004). Studies on molecular mechanisms underlying high pressure adaptation of *α*-actin from deep-sea fish. *Bulletin of Fisheries Research & Development Agency*.

[9] Morita T (2008). Comparative sequence analysis of myosin heavy chain proteins from congeneric shallow- and deep-living rattail fish (genus *Coryphaenoides*). *The Journal of Experimental Biology*.

[10] Morita T (2010). High-pressure adaptation of muscle proteins from deep-sea fishes, *Coryphaenoides yaquinae* and *C. armatus*. *Annals of the New York Academy of Sciences*.

[11] Hourdez S, Weber RE (2005). Molecular and functional adaptations in deep-sea hemoglobins. *Journal of Inorganic Biochemistry*.

[12] Tucker DW (1956). Studies on Trichiuroid fishes—3. A preliminary revision of the family Trichiuridae. *Bulletin of the British Museum (Natural History) Zoology*.

[13] Martins MR, Leite AM, Nunes ML Peixe-espada-preto. Algumas notas ácerca da pescaria do peixe-espada-preto.

[14] FAO Fishstat.

[15] Machete M, Morato T, Menezes G (2011). Experimental fisheries for black scabbardfish (*Aphanopus carbo*) in the Azores, Northeast Atlantic. *ICES Journal of Marine Science*.

[16] ICES (2008). Report of the Working Group on the Biology and Assssment of Deep-Sea Fisheries Resources (WGDEEP).

[17] Figueiredo I, Bordalo-Machado P, Reis S (2003). Observations on the reproductive cycle of the black scabbardfish (*Aphanopus carbo* Lowe, 1839) in the NE Atlantic. *ICES Journal of Marine Science*.

[18] Morales-Nin B, Sena-Carvalho D (1996). Age and growth of the black scabbard fish (*Aphanopus carbo*) off Madeira. *Fisheries Research*.

[19] Longmore C, Trueman CN, Neat F (2014). Ocean-scale connectivity and life cycle reconstruction in a deep-sea fish. *Canadian Journal of Fisheries and Aquatic Sciences*.

[20] Stefanni S, Knutsen H (2007). Phylogeography and demographic history of the deep-sea fish *Aphanopus carbo* (Lowe, 1839) in the NE Atlantic: vicariance followed by secondary contact or speciation?. *Molecular Phylogenetics and Evolution*.

[21] Nagaraj SH, Gasser RB, Ranganathan S (2007). A hitchhiker’s guide to expressed sequence tag (EST) analysis. *Briefings in Bioinformatics*.

[22] Hudson ME (2008). Sequencing breakthroughs for genomic ecology and evolutionary biology. *Molecular Ecology Resources*.

[23] Ellegren H (2008). Sequencing goes 454 and takes large-scale genomics into the wild. *Molecular Ecology*.

[24] Bettencourt R, Pinheiro M, Egas C (2010). High-throughput sequencing and analysis of the gill tissue transcriptome from the deep-sea hydrothermal vent mussel Bathymodiolus azoricus. *BMC Genomics*.

[25] Lesniewski RA, Jain S, Anantharaman K, Schloss PD, Dick GJ (2012). The metatranscriptome of a deep-sea hydrothermal plume is dominated by water column methanotrophs and lithotrophs. *The ISME Journal*.

[26] Wu J, Gao W, Zhang W, Meldrum DR (2011). Optimization of whole-transcriptome amplification from low cell density deep-sea microbial samples for metatranscriptomic analysis. *Journal of Microbiological Methods*.

[27] Shi Y, McCarren J, Delong EF (2012). Transcriptional responses of surface water marine microbial assemblages to deep-sea water amendment. *Environmental Microbiology*.

[28] Stefanni S, Bettencourt R, Knutsen H, Menezes G (2009). Rapid polymerase chain reaction-restriction fragment length polymorphism method for discrimination of the two Atlantic cryptic deep-sea species of scabbardfish. *Molecular Ecology Resources*.

[29] Margulies M, Egholm M, Altman WE (2005). Genome sequencing in microfabricated high-density picolitre reactors. *Nature*.

[30] Altschul SF, Gish W, Miller W, Myers EW, Lipman DJ (1990). Basic local alignment search tool. *Journal of Molecular Biology*.

[31] Thompson JD, Gibson TJ, Plewniak F, Jeanmougin F, Higgins DG (1997). The CLUSTAL X windows interface: flexible strategies for multiple sequence alignment aided by quality analysis tools. *Nucleic Acids Research*.

[32] Saitou N, Nei M (1987). The neighbor-joining method: a new method for reconstructing phylogenetic trees. *Molecular Biology and Evolution*.

[33] Felsenstein J (1985). Confidence limits on phylogenies: an approach using the bootstrap. *Evolution*.

[34] Yang Z (2007). PAML 4: a program package for phylogenetic analysis by maximum likelihood. *Molecular Biology and Evolution*.

[35] Prashanth Kumar S, Meenatchi M (2011). Virtual quantification of protein stability using applied kinetic
and thermodynamic parameters. *IIOAB Letters*.

[36] Thiel T, Michalek W, Varshney RK, Graner A (2003). Exploiting EST databases for the development and characterization of gene-derived SSR-markers in barley (Hordeum vulgare L.). *Theoretical and Applied Genetics*.

[37] Rozen S, Skaletsky H (2000). Primer3 on the WWW for general users and for biologist programmers.. *Methods in Molecular Biology*.

[38] Lee B-Y, Howe AE, Conte MA (2010). An EST resource for tilapia based on 17 normalized libraries and assembly of 116,899 sequence tags. *BMC Genomics*.

[39] Drazen JC, Seibel BA (2007). Depth-related trends in metabolism of benthic and benthopelagic deep-sea fishes. *Limnology and Oceanography*.

[40] Sullivan KM, Somero GN (1980). Enzyme activities of fish skeletal muscle and brain as influenced by depth of occurrence and habits of feeding and locomotion. *Marine Biology*.

[41] Siebenaller JF, Somero GN, Haedrich RL (1982). Biochemical characteristics of macrourid fishes differing in their depths of distribution. *The Biological Bulletin*.

[42] Merrit TJS, Quattro JM (2003). Evolution of the vertebrate cytosolic malate dehydrogenase gene family: duplication and divergence in actinopterygian fish. *Journal of Molecular Evolution*.

[43] Ikkai T, Ooi T (1966). The effects of pressure on F-G transformation of actin. *Biochemistry*.

[44] Verde C, Balestrieri M, de Pascale D, Pagnozzi D, Lecointre G, Di Prisco G (2006). The oxygen transport system in three species of the boreal fish family Gadidae: molecular phylogeny of hemoglobin. *The Journal of Biological Chemistry*.

[45] Him HJ, Steif C, Vogl T (1993). Fundamentals of protein stability. *Pure and Applied Chemistry*.

[46] Hoekstra HE, Coyne JA (2007). The locus of evolution: evo devo and the genetics of adaptation. *Evolution*.

[47] Yancey PH, Fyfe-Johnson AL, Kelly RH (2001). Trimethylamine oxide counteracts effects of hydrostatic pressure on proteins of deep-sea teleosts. *Journal of Experimental Zoology*.

[48] Yancey PH, Blake WR, Conley J (2002). Unusual organic osmolytes in deep-sea animals: adaptations to hydrostatic pressure and other perturbants. *Comparative Biochemistry and Physiology A: Molecular & Integrative Physiology*.

[49] Yancey PH, Rhea MD, Kemp KM, Bailey DM (2004). Trimethylamine oxide, betaine and other osmolytes in deep-sea animals: depth trends and effects on enzymes under hydrostatic pressure. *Cellular and Molecular Biology*.

[50] Prunet P, Cairns MT, Winberg S, Pottinger TG (2008). Functional genomics of stress responses in fish. *Reviews in Fisheries Science*.

