# Supplementary material for: Transcriptome of the Deep-Sea Black Scabbardfish, Aphanopus carbo (Perciformes: Trichiuridae): Tissue-Specific Expression Patterns and Candidate Genes Associated to Depth Adaptation
Source: Int J Genomics. 2014 Sep 17;2014:267482. doi: 10.1155/2014/267482 (PMC4182897; doi:10.1155/2014/267482)
Supplement: Supplementary file 1 — As supplementary material we included: amplification plots of all genes selected for this study in different tissue from Aphanopus carbo (Figure S1); a summary table of contig frequency in all tissues for those genes associated to depth not listed in Table 3 (Table S1); details on outputs related to the selection models for four teleost genes associated to depth (Table S2); values of Gibbs free energy calculations in terms of kinetic and thermodynamic quantities for all depth-related genes (Table S3); and the list of EST-SSR markers (Table S4). [file 267482.f1.pdf]

Supplementary material

Fig. S1. Amplification plots of all genes selected for this study in different tissue from *Aphanopus carbo*. The cycle numbers are plotted against the change in fluorescence units expressed in logarithmic scale, and the horizontal line represents the threshold line. S = spleen, B = brain, H = heart, G = gonad, L = liver, M = muscle, * = expression in the brain was below detection and ** = expression in the gonads was below detection.

Tab. S1. Summary table of contig frequency in all tissues for those genes associated to depth not listed in table 3.

Tab. S2. Site-specific selection models for four teleot genes associated to depth. The likelihood (*lnL*) values and the respective parameters are shown for the two models, M1 (codons under negative or neutral selection) and M2 (which includes codons under positive selection). *K* = free parameters; ω = ratio of nonsynonymous *vs.* synonymous substitutions for each class defined in the models and *p* = proportion of codon sites belonging to each class of the model tested.

Tab. S3. Values of Gibbs free energy calculations in terms of kinetic and thermodynamic quantities for all depth-related genes. PS = protein stability; GE = Gibbs free energy; ΔG = Normalized Gibbs energy change. Taxon coding include the first two letters for the genus followed by three letters for the species name (see Tab. 6).

Tab. S4. List of EST-SSR markers by locus, Genbank accession number, type of motif, sets of primer sequences with relative melting temperatures, expected size of the PCR amplified product and tissues in which the gene targets are expressed (a tissue code within brackets indicates that ESTs were significantly more abundant). Loci were separated by alternating bold and regular

character styles. S = spleen, B = brain, H = heart, G = gonad, L = liver, M = muscle.

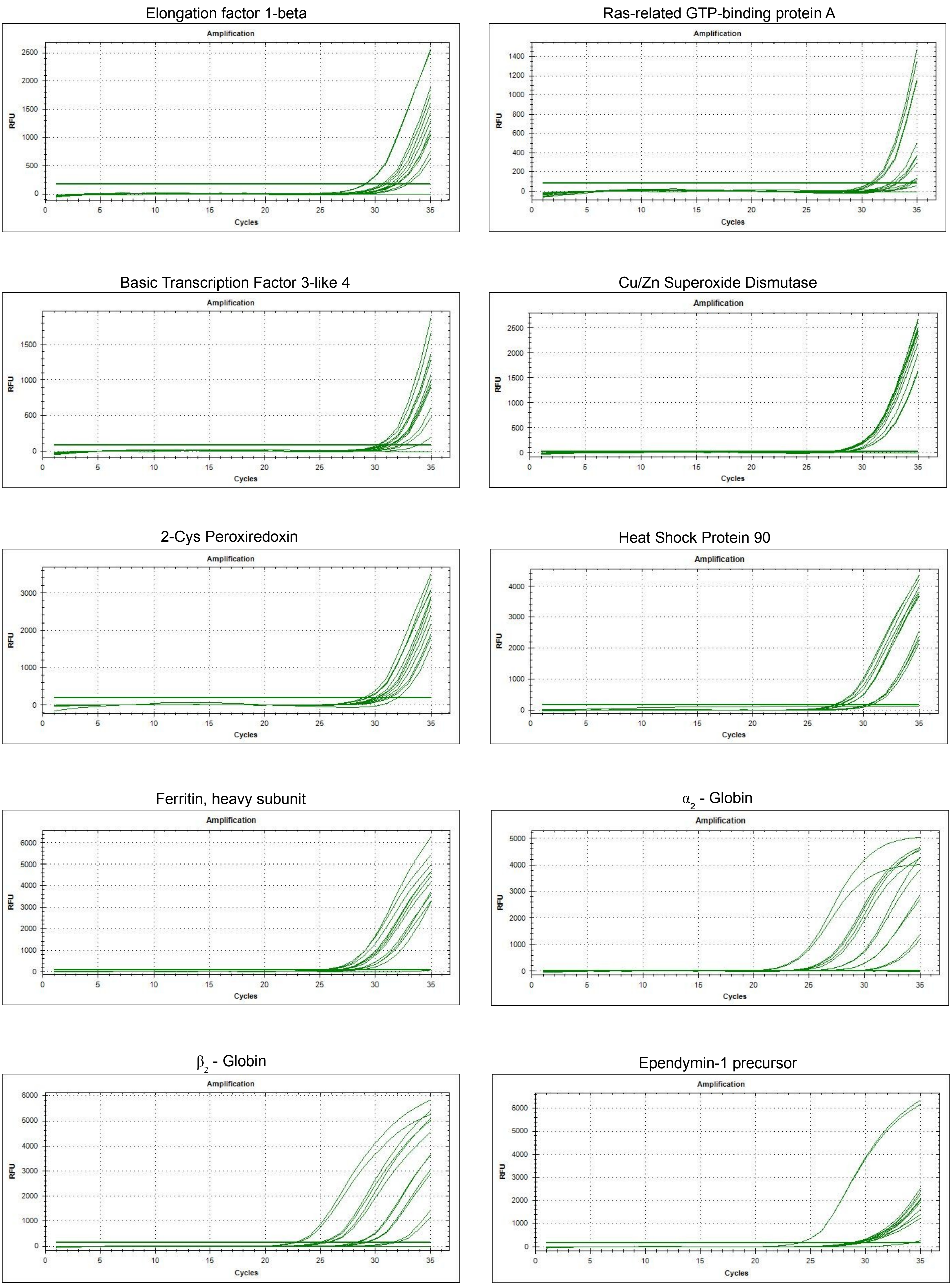

Elongation factor 1-beta
Ras-related GTP-binding protein A
Basic Transcription Factor 3-like 4
Cu/Zn Superoxide Dismutase
2-Cys Peroxiredoxin
Heat Shock Protein 90
Ferritin, heavy subunit
α_2 - Globin
β_2 - Globin
Ependymin-1 precursor
Amplification
RFU
Cycles

Fig. S1

Fatty acid-binding protein, brain

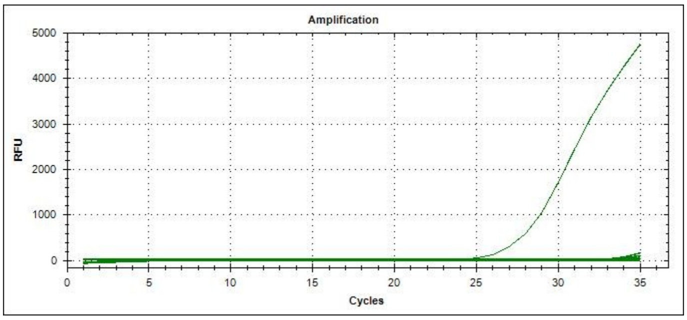

CD63-like protein Sm-TSP-2

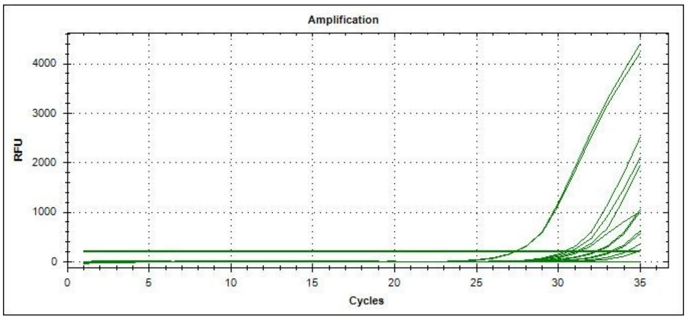

Tropomyosin 4 isoform 1

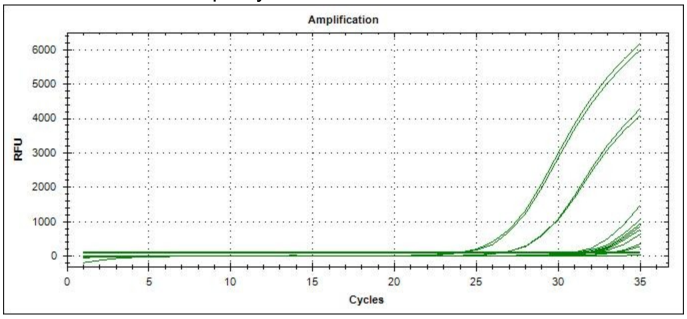

C-Myc-binding protein

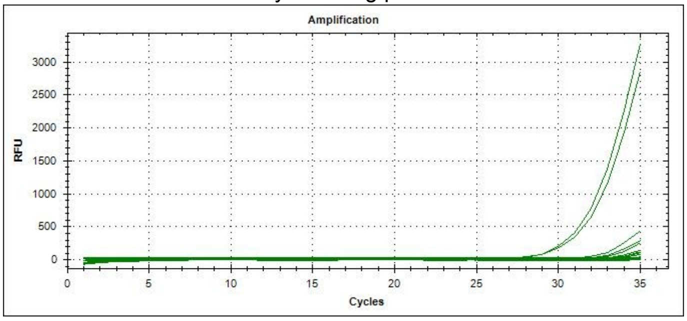

Cathepsin S

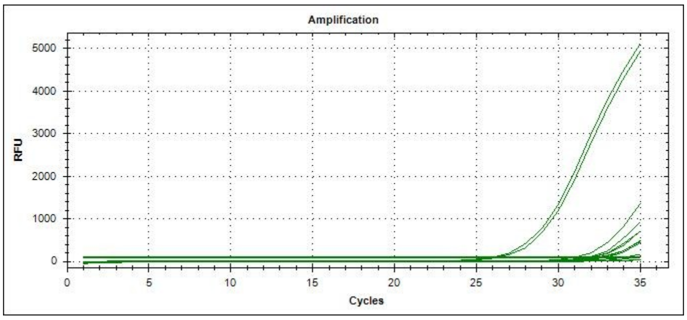

Transferrin

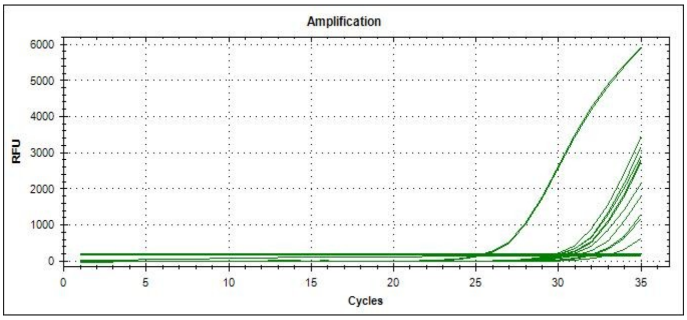

Warm Temperature Acclimation related-like 65kDa protein

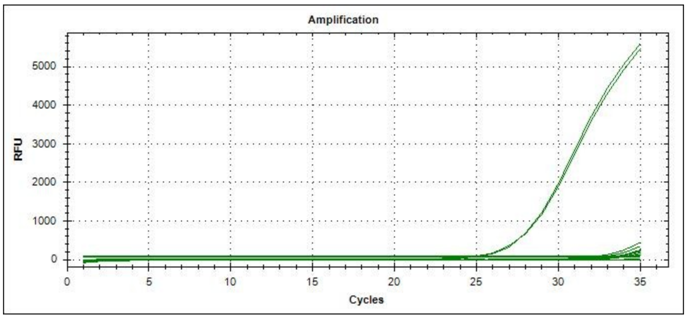

Betaine Homocysteine S-methyltansferase

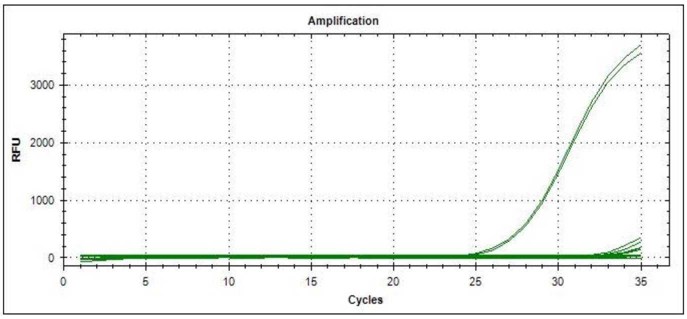

FUCL1 Fucolectin-1

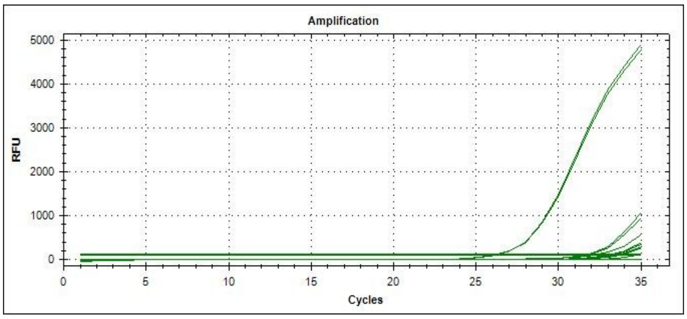

Aldolase B

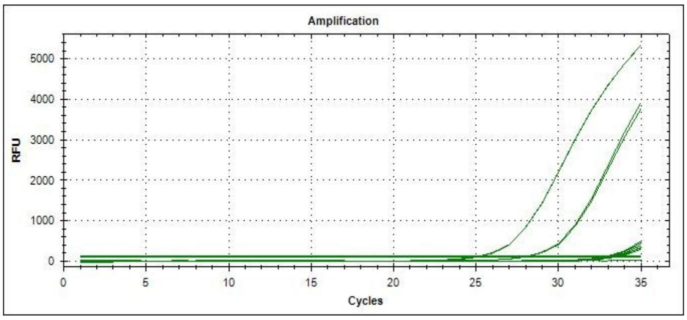

Fig. S1

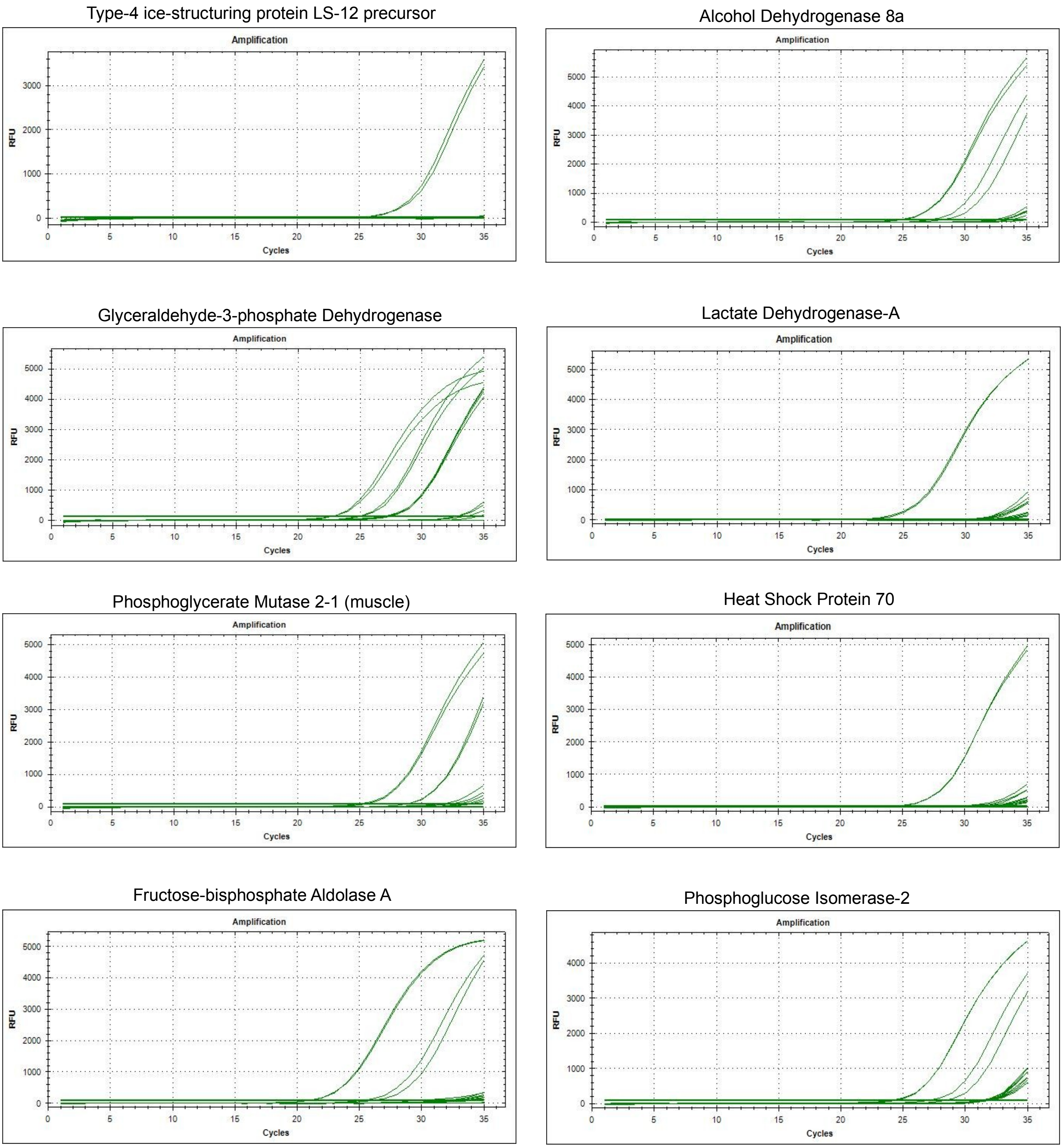
Type-4 ice-structuring protein LS-12 precursor
Alcohol Dehydrogenase 8a
Glyceraldehyde-3-phosphate Dehydrogenase
Lactate Dehydrogenase-A
Phosphoglycerate Mutase 2-1 (muscle)
Heat Shock Protein 70
Fructose-bisphosphate Aldolase A
Phosphoglucose Isomerase-2
Amplification
RFU
Cycles

Fig. S1

Enzyme	Contig code	Brain	Heart	Gonad	Liver	Muscle	Spleen
LDH-B	isotig01423	3	241	0	1	2	5
MDHc-A	isotig01010	25	86	2	26	0	5
MDHc-B*	isotig01731	0	3	35	0	10	1
Actin 1	isotig01459	1	40	0	0	1591	1
Actin 2a	isotig01561	0	3	0	0	501	0
MyHC	isotig01394**	0	1	0	0	2199	0

*hypothetical
**inclusive of the complementary sequences isotig02568 and isotig01834

Tab S1.

Gene	Model	*ω*	*lnL*	*K*	*p*
LDH-A	M1	0.020 / 1.000	-3090.2583	17	0.881 / 0.119
	M2	0.020 / 1.000 / 9.952	-3090.2583	19	0.881 / 0.119 / 0.000
LDH-B	M1	0.032 / 1.000	-2981.1259	15	0.948 / 0.052
	M2	0.032 / 1.000 / 5.558	-2981.1259	17	0.948 / 0.052 / 0.000
MDHc	M1	0.023 / 1.000	-2636.4461	11	0.944 / 0.056
	M2	0.023 / 1.000 / 12.563	-2636.3758	13	0.945 / 0.052 / 0.003
ACTA1	M1	0.001 / 1.000	-2594.7798	17	0.976 / 0.024
	M2	0.006 / 1.000 / 1.000	-2594.7795	19	0.976 / 0.007 / 0.016

Tab S2.

LDH A	Kinetic calculations		Thermodynamic calculations		ΔG (cal/mol)
	PS (Kcal/mol)	GE (Kcal/mol)	PS (Kcal/mol)	GE (KJ/mol)	
Ac_car	0.3705	252.95	0.3645	-5780.92	6.0
Ch_cau	0.3614	248.20	0.3675	-5805.17	-6.1
Ch_ras	0.3746	254.41	0.3595	-5655.25	15.1
Rh_nic	0.3735	256.85	03735	-5914.41	0
Cy_car	0.3825	264.37	03554	-5637.11	27.1
Fu_het	0.3735	257.62	0.3584	-5679.62	15.1
No_cor	0.3716	252.12	0.3595	-5655.25	12.1
Sp_idi	0.3765	257.86	0.3584	-5686.14	18.1

LDH B	Kinetic calculations		Thermodynamic calculations		ΔG (cal/mol)
	PS (Kcal/mol)	GE (Kcal/mol)	PS (Kcal/mol)	GE (KJ/mol)	
Ac_car	0.3563	250.74	0.3563	-5758.11	0
Co_arm	0.3743	261.18	0.3383	-5495.30	36.0
Fu_het	0.3713	260.54	0.3503	-5669.52	21.0
Ga_mor	0.3754	261.53	0.3363	-5415.66	39.1
La_cal	0.3683	259.15	0.3383	-5481.69	30.0
Me_mer	0.3754	261.53	0.3393	-5467.26	36.1
Po_ret	0.3650	203.71	0.3574	-4607.34	7.6
Tr_mur	0.3743	263.18	0.3353	-5433.19	39.0

MDHc	Kinetic calculations		Thermodynamic calculations		ΔG (cal/mol)
	PS (Kcal/mol)	GE (Kcal/mol)	PS (Kcal/mol)	GE (KJ/mol)	
Ac_car	0.3904	279.66	0.3784	-6234.9	12.0
Or_lat	0.3904	281.69	0.3724	-6131.24	18.0
Os_mor	0.3844	276.93	0.3784	-6200.19	6.0
Sa_sal	0.3844	275.90	0.3814	-6294.07	3.0
Sp_idi	0.3994	283.63	0.3754	-6166.56	24.0

Actin 1	Kinetic calculations		Thermodynamic calculations		ΔG (cal/mol)
	PS (Kcal/mol)	GE (Kcal/mol)	PS (Kcal/mol)	GE (KJ/mol)	
Ac_car	0.3973	312.76	0.3413	-6359.41	56.0
Co_acr	0.3973	312.76	0.3413	-6359.41	56.0
Co_cin	0.3973	312.76	0.3413	-6359.41	56.0
Cy_car	0.3973	312.76	0.3413	-6369.16	56.0
Or_lat	0.3973	312.76	0.3413	-6359.41	56.0
Pl_azo	0.3973	312.76	0.3413	-6359.41	56.0
Sc_sco	0.3973	312.76	0.3413	-6359.41	56.0
Si_chu	0.3873	312.76	0.3413	-6359.41	56.0
Sp_aur	0.3893	305.17	0.3493	-6520.33	40.0
Sp_idi	0.3946	310.27	0.3387	-6309.15	55.9

Actin 2a	Kinetic calculations		Thermodynamic calculations		ΔG (cal/mol)
	PS (Kcal/mol)	GE (Kcal/mol)	PS (Kcal/mol)	GE (KJ/mol)	
Ac_car	0.3973	312.63	0.3387	-6307.82	58.6

Co_acr	0.3973	312.76	0.3413	-6349.67	56.0
Co_arm	0.3973	312.76	0.3413	-6349.67	56.0
Co_cin	0.3973	312.76	0.3413	-6349.67	56.0
Co_yaq	0.3973	312.76	0.3413	-6349.67	56.0

**Actin 2b**	**Kinetic calculations**		**Thermodynamic calculations**		**ΔG (cal/mol)**
	**PS (Kcal/mol)**	**GE (Kcal/mol)**	**PS (Kcal/mol)**	**GE (KJ/mol)**	
Co_arm	0.3973	312.26	0.3467	-6425.12	50.6
Co_yaq	0.3947	309.97	0.3440	-6373.53	50.7

**Hb-A**	**Kinetic calculations**		**Thermodynamic calculations**		**ΔG (cal/mol)**
	**PS (Kcal/mol)**	**GE (Kcal/mol)**	**PS (Kcal/mol)**	**GE (KJ/mol)**	
Ac_car	0.4437	134.93	0.4085	-2829.51	35.2
Ar_gla	0.4507	142.71	0.4014	-2788.9	49.3
Bo_sai	0.4507	142.21	0.3944	-2729.06	56.3
Ga_mor	0.4014	126.31	0.4085	-2829.51	-7.1
No_ang	0.3901	123.9	0.4043	-2794.26	-14.2
Ta_rub	0.4014	126.8	0.3732	-2639.74	28.2

**Hb-B**	**Kinetic calculations**		**Thermodynamic calculations**		**ΔG (cal/mol)**
	**PS (Kcal/mol)**	**GE (Kcal/mol)**	**PS (Kcal/mol)**	**GE (KJ/mol)**	
Ac_car	0.3836	124.0	0.3425	-2535.55	41.1
An_fim	0.3904	121.84	0.3493	-2523.34	41.1
Bo_sai	0.4110	132.06	0.3356	-2463.14	75.4
Ga_acu	0.4041	126.23	0.3356	-2418.82	68.5
No_ang	0.3836	118.93	0.3699	-2643.42	13.7

Tab. S3

Locus	GB acc nr	Repeat motif	FORWARD PRIMER1 (5'-3')	Tm(ºC)	REVERSE PRIMER1 (5'-3')	Tm(ºC)	size (bp)	Tissue
Ac1400		(GATG)5	GACTATGTCTCTCGGCTGGC	60.0	CAGAATGACTGCATCAGGGA	59.8	186	B/G/L/M/S
			AGGAAGAGCTGGACGAAGTG	59.6	CAGAATGACTGCATCAGGGA	59.8	143	
**Ac1461**		**(ACA)9**	**TTGTTGCTCATGTTTCGTCC**	**59.7**	**GAGGAGACCATCAAGAAGCG**	**60.0**	**252**	**B/G/H/(M)**
			**TTGTTGCTCATGTTTCGTCC**	**59.7**	**CATCGTCAGGAAGGTGGACT**	**60.1**	**175**	
Ac1465		(CA)13	GTTGGTCCGAGTTGGAGTTC	59.6	CTCCAAACACCAGGGACTGT	60.0	171	(G)/H/M
			GTTGGTCCGAGTTGGAGTTC	59.6	GTCCCTGGACTCTGGAACAA	60.1	197	
			GTTGGTCCGAGTTGGAGTTC	59.6	CTTCTAAACACGACGCCTCC	59.9	257	
**Ac0512**		**(ATCA)22**	**TTACTCAGCAACATGCCAGC**	**60.0**	**GAGCTTCTATCCTCAGGCCA**	**59.5**	**265**	**B/G/H/(L)/S**
			**GCAGCCTCTGTTACTCAGCA**	**59.3**	**GAGCTTCTATCCTCAGGCCA**	**59.5**	**275**	
			**TTACTCAGCAACATGCCAGC**	**60.0**	**TCCTGAATGAGGTCACTGCTT**	**59.9**	**239**	
Ac0513		(ATCA)5t(TCAA)12	TTACTCAGCAACATGCCAGC	60.0	GCACAGAGAGGCTCAGGAAG	60.3	264	B/G/H/(L)/S
			TTACTCAGCAACATGCCAGC	60.0	GAGCTTCTATCCTCAGGCCA	59.5	245	
**Ac0848**		**(TG)12**	**GGAGCCAACCTGTTGAGTGT**	**60.2**	**ACACGTTGTGTTTAGCAGCG**	**60.0**	**154**	**B/(G)/(H)/L/M/S**
			**AGTAGTGAAGGGTGTTGGCG**	**60.2**	**ACACGTTGTGTTTAGCAGCG**	**60.0**	**131**	
			**GGAGCCAACCTGTTGAGTGT**	**60.2**	**GGAACCTTCTCACAGGCAAG**	**59.8**	**124**	
Ac1648		(CAA)8	GAAAGCATGGTGGCTCCTAA	60.2	TGTGGTCTTGCGATGAAGAG	60.0	168	B/H/L/M
			GCGCCGAGGACACTTTATTA	60.2	TGTGGTCTTGCGATGAAGAG	60.0	245	
			TAAAAGAAGTCCGCGGTCAC	60.2	TGTGGTCTTGCGATGAAGAG	60.0	208	
**Ac1657**		**(TCAA)5**	**TGATGTCCCTTGTTGTGCAT**	**60.0**	**AGGCTGACAAACCGTCTGAT**	**59.7**	**256**	**B/G/H/L/M/S**
			**GGATTCTGTCGATGCCTGTT**	**60.1**	**AGGCTGACAAACCGTCTGAT**	**59.7**	**173**	
			**GCGCTGTTATTGATGTCCCT**	**60.1**	**AGGCTGACAAACCGTCTGAT**	**59.7**	**266**	
Ac1684		(TTG)9	TGTTTAATGCAGTGAGCGGA	60.4	GTCCAACGTTTGGTCGTTTT	59.9	167	B/G/H/L/M/S
			TGTTTAATGCAGTGAGCGGA	60.4	AAATGTGTGCATGCAGGAAG	59.7	189	
			AGCAGCTGGAGGACGTTACA	61.0	GTCCAACGTTTGGTCGTTTT	59.9	262	
**Ac1692**		**(CTC)8**	**CCTCCTCCATCATCTCCTGA**	**60.2**	**AGGAGGAATCAGCCAAGGTT**	**60.1**	**123**	**B**
			**TCCTCCTCCATCATCTCCTG**	**60.2**	**AGGAGGAATCAGCCAAGGTT**	**60.1**	**124**	
			**CCTCCTCCATCATCTCCTGA**	**60.2**	**TTCCTCCTCCTCCTCTCCTC**	**59.9**	**239**	
Ac0944		(GAG)8	CAGCTGTAACTCCAGCTCCC	60.0	TCTTCATCACTGCCGAACAG	60.0	151	B/G/H/L/M/S
			CAGCTGTAACTCCAGCTCCC	60.0	GCGTACGCTTTCAACCTCTC	60.0	196	
			CAGCTGTAACTCCAGCTCCC	60.0	GCCGAACAGGTCAATATCGT	60.0	140	
**Ac1700**		**(AGC)10**	**AGCAACAAGCACTCCCTCAT**	**59.9**	**TGCTCGTACATGTCCCTCTG**	**59.9**	**236**	**B/G/H/L/M**
			**AGCAACAAGCACTCCCTCAT**	**59.9**	**CCCCACTCCATGTCATTCTT**	**59.8**	**278**	
Ac1735		(GT)13	TTCTTCCCACCGATAACCTG	59.9	CACACACAAAAGTTGGACCG	60.0	200	B/G/L/M/S
			CCCGGAGTCTGGATGAATTA	59.9	GTTTATTTTTGGCGGAGCAA	60.1	271	
			TTCTTCCCACCGATAACCTG	59.9	TTTATTTTTGGCGGAGCAAG	60.2	150	
**Ac0945**		**(GAG)8**	**CAGCTGTAACTCCAGCTCCC**	**60.0**	**TCTTCATCACTGCCGAACAG**	**60.0**	**151**	**B/G/H/L/M/S**
			**CAGCTGTAACTCCAGCTCCC**	**60.0**	**GCGTACGCTTTCAACCTCTC**	**60.0**	**196**	

Locus	GB acc nr	Repeat motif	FORWARD PRIMER1 (5'-3')	Tm(ºC)	REVERSE PRIMER1 (5'-3')	Tm(ºC)	size (bp)	Tissue
Ac1773		(CA)14	TTCTGGTGGTGGGTTTTACC	59.7	CATGTCACGTTTTATTGCCC	58.9	100	B/G/H/L/M/S
			AAAGCACCAAAGTGCTCTTCA	60.0	TTGCCCAACTCTAGGAGTGC	60.4	177	
			TCTCCAATGATGGTGAACACA	60.0	TTGCCCAACTCTAGGAGTGC	60.4	255	
**Ac1817**		**(GT)20**	**CTTGACACACAAACACACACTCA**	**59.7**	**AACTGGCAGGACAAGCTGTT**	**59.9**	**208**	**B/G/L/M/S**
			**GCTCAGGTATTTTCAGTGCAGTC**	**60.3**	**GAGATCGCGACCTACAGGAG**	**60.0**	**224**	
Ac1860		(AATC)10	GACCCAAACTGAAGCAGCTC	60.0	ACAATCGGCTCGTGTCATTT	60.5	186	B/G/H/L/M/S
			AAAACACAAAGGATGCTGGG	60.0	ACAATCGGCTCGTGTCATTT	60.5	211	
**Ac1860**		**(AC)17**	**CGGCTGGAAAATCAATCAAT**	**59.9**	**CGTCCTACAAACCACCGACT**	**60.0**	**255**	**B/G/H/L/M/S**
			**AATCAATCAATCAATCCGGC**	**59.7**	**CGTCCTACAAACCACCGACT**	**60.0**	**222**	
			**CGGCTGGAAAATCAATCAAT**	**59.9**	**TTTGCATGTTCTGTTCGTCC**	**59.7**	**270**	
Ac2043		(TGTA)10	GGAGAGGCTGTCAGACTTCAA	59.6	CCCGAATATGAAGCCAAAAC	59.4	272	(H)/L
**Ac2060**		**(TCA)8**	**CAGCGTGAGGATGATGAAAA**	**59.8**	**ATGACGACGACGATGATGAC**	**59.5**	**272**	**M**
			**CAGCGTGAGGATGATGAAAA**	**59.8**	**GACGATGATGACGACGATGA**	**60.7**	**243**	
Ac2117		(ATT)11	AGGAGAGTGAAGTTTCCGCA	60.0	CAATTAGGCCATCTCACCAC	58.0	237	B/G/H/L/M
**Ac2124**		**(AC)13**	**ACACACACACGCACACACAC**	**60.2**	**GAGCCTGCTAGGAAAGAGCC**	**60.6**	**101**	**B/G/L/M/S**
Ac2130		(TG)17	GAGGCCTACCCCCAGTAAAG	60.0	AGTCATATCTGGTCACAACACCA	59.4	233	B/G/H/L/M/S
**Ac0582**		**(GT)20**	**TCTACAGTCAATCCCCACCC**	**59.8**	**AGGTACGAGACGCTCCTGAA**	**60.0**	**217**	**B/G/H/L/M/S**
			**TCTACAGTCAATCCCCACCC**	**59.8**	**GGATGAGGCCAAAGCTACAG**	**59.8**	**275**	
Ac0617		(AAAT)5	CAAGCAAGACCAGTGCAAAA	60.0	CGGTACCAACCATGTGATGA	60.2	232	H
			AGCATTAGGGCTGTCAATCG	60.2	CGGTACCAACCATGTGATGA	60.2	113	
**Ac2218**		**(ATCT)6**	**GAGCCCTGTGCTACTTTTGC**	**60.0**	**GAGCTCCAGTGATGGGAGAG**	**59.9**	**275**	**B/G/H/M/S**
			**TTTCTGTTTGCCTTCATCCC**	**60.1**	**GAGCTCCAGTGATGGGAGAG**	**59.9**	**227**	
Ac0618		(AAAT)5	CAAGCAAGACCAGTGCAAAA	60.0	CGGTACCAACCATGTGATGA	60.2	232	H
			AGCATTAGGGCTGTCAATCG	60.2	CGGTACCAACCATGTGATGA	60.2	113	
**Ac2335**		**(TGT)9**	**TTGACAGCTCGTTCTCCTGA**	**59.7**	**CCTCAAAACAAGAAAGCCGA**	**60.4**	**145**	**G/H/M/S**
Ac1036		(TGA)8	CAAGCCTCGAAGACGTAAGG	60.0	AGTTTCTGCTTTGGCACAGG	60.4	162	H/L
			CTGATGGTCATCCTGGTCCT	59.9	AGTTTCTGCTTTGGCACAGG	60.4	218	
			CAAGCCTCGAAGACGTAAGG	60.0	GGGTAGCAAAGAGCTCGTCA	60.5	268	
**Ac2385**		**(ATG)11**	**GACATGATGCAGAGGCAGTG**	**60.4**	**CCTCTGACCCTTCCTAACCC**	**59.9**	**174**	**B/M**
			**GACATGATGCAGAGGCAGTG**	**60.4**	**TGTCCAATGAGCAACCAGAG**	**59.8**	**236**	
Ac2551		(TG)12	CAAAGATGGCAAAGAGCACA	60.0	TGCAAGGACAAGAAGGAAGG	60.4	233	B/H/L/S
			CAAGAAGCACAAGTCCGACA	60.0	TGCAAGGACAAGAAGGAAGG	60.4	272	
			CCGACAGTAACGACGAGTCA	59.9	TGCAAGGACAAGAAGGAAGG	60.4	258	
**Ac2617**		**(GCA)8**	**AGCCGCTGTACTGAACAGGT**	**59.9**	**TTAAAAGGTGCTGGGCAGAT**	**59.7**	**151**	**B**
			**CTGGCTGGAGGTGAAGTGTT**	**60.3**	**TTAAAAGGTGCTGGGCAGAT**	**59.7**	**125**	
Ac0432		(AAT)9	TTACAGGCTGGTGACAGGTG	59.7	GGACTACAGTGAGAAGCCGC	60.0	233	L
			GGTTTGCTGCAGTTGGTCTT	60.3	GGACTACAGTGAGAAGCCGC	60.0	214	
			TTACAGGCTGGTGACAGGTG	59.7	ATGAGGGACGGACTGACAAG	60.1	252	
**Ac2769**		**(ATG)13**	**TAGAATTTGGTGGTGCTCCC**	**59.9**	**TGAATCATTCCCCTGCATCT**	**60.4**	**149**	**L**

Locus	GB acc nr	Repeat motif	FORWARD PRIMER1 (5'-3')	Tm(ºC)	REVERSE PRIMER1 (5'-3')	Tm(ºC)	size (bp)	Tissue
Ac2832		(TG)12	TCTGTGCTTCAGTGTGTCAGC	60.2	TCAGGCACCAACAGTTTTCA	60.3	272	(L)/H
**Ac2911**		**(GCT)9**	**TCAGTTCCTTCGTCAGGGAT**	**59.7**	**CTTCTGGATGAGGAGAACGC**	**60.0**	**221**	**B/G/H/L/M**
Ac1037		(TGA)8	CAAGCCTCGAAGACGTAAGG	60.0	AGTTTCTGCTTTGGCACAGG	60.4	162	H/L
			CTGATGGTCATCCTGGTCCT	59.9	AGTTTCTGCTTTGGCACAGG	60.4	218	
			CAAGCCTCGAAGACGTAAGG	60.0	GGGTAGCAAAGAGCTCGTCA	60.5	242	
**Ac3081**		**(AAAT)5**	**TATGGAGGACTGGAACTGCC**	**60.1**	**GCATGTGGTCACTACAAAGCA**	**59.8**	**262**	**G/L**
Ac0516		(GT)61	AACCTGCAGAAAGCCAAAGA	60.0	CAACCACAACACTGCTGCTT	59.9	219	B/G/H/L/M/S
			GTCGAACACAACCTGCAGAA	59.9	CAACCACAACACTGCTGCTT	59.9	228	
**Ac0517**		**(GT)79**	**CATTTTCCATCTGGACTGGC**	**60.5**	**CAACCACAACACTGCTGCTT**	**59.9**	**258**	**B/G/H/L/M/S**
Ac3230		(TG)14	TGACGTGATGCTGGATGAAT	60.1	GGGGATTGTGGAGCATAAAT	58.7	263	H/L
**Ac3316**		**(TAT)10**	**GCTTCAGAAAAGACCTGCGT**	**59.6**	**GGCATGATCCCTTAACCTGA**	**59.9**	**222**	**G/L**
			GCTTCAGAAAAGACCTGCGT	59.6	AATACGTGAAGCAAGGGACG	60.1	141	
**Ac3266**		**(ATG)10**	**GTATTGATCCTCGTCCTCCG**	**59.5**	**AGATAACCAATGCAGGCAGC**	**60.2**	**194**	**B/G/H/M/S**
			**TATCCCGTTAACGACACGCT**	**60.5**	**AGATAACCAATGCAGGCAGC**	**60.2**	**260**	
Ac3328		(CA)14	TGAAGACCAAACGCTGTTAGC	60.4	TTTGTTGTGTGCATGTGTGTG	60.1	182	B/G/M/S
**Ac3511**		**(AGAC)5**	**TATGCTGTGCAGGAGGACAC**	**59.9**	**GTGCCTGGATGTCGAAGTTT**	**60.1**	**102**	**G/L/M**
			**TATGCTGTGCAGGAGGACAC**	**59.9**	**GAGAAGACCCACTGCAGGAC**	**59.8**	**236**	
			**TATGCTGTGCAGGAGGACAC**	**59.9**	**AAGGCCAGGAAGGAGAAGAC**	**59.8**	**248**	
Ac3540		(CA)15	ACTTGATTTTCCTGCACCGT	59.6	TTACTCCACCAACCTGAGCG	61.2	100	L
**Ac0714**		**(ACA)8**	**CTCAGGGGGCTTTACAGTCT**	**58.4**	**CGTTACTGTGGGGGATTGAC**	**60.2**	**279**	**B/G/L/M/S**
Ac3684		(GAAAT)5	AGCAGTCTACACAAAGCTGGC	59.7	GAGCGAACAGAAGAACGACC	60.0	163	M
			AGCAGTCTACACAAAGCTGGC	59.7	CCATCACACGAAACAACGAC	60.0	220	
**Ac3766**		**(TGT)9**	**GTTTCTGTGCTCGAAGGACC**	**59.9**	**AAGTTTCCTCACATGCCAGC**	**60.3**	**135**	**G/H/M**
			**TATTTCTTGAAGGGAGGGGG**	**60.3**	**AAGTTTCCTCACATGCCAGC**	**60.3**	**251**	
			**ACCAGCTGGATGAAGTCCTG**	**60.3**	**AAGTTTCCTCACATGCCAGC**	**60.3**	**200**	
Ac0583		(GT)15	TCTACAGTCAATCCCCACCC	59.8	ATCAGGTTTCTTCCCTCCGT	59.9	231	B/G/H/L/M/S
			TCTACAGTCAATCCCCACCC	59.8	AAGAACTTCTGCAAGCCTGG	59.6	170	
			TCTACAGTCAATCCCCACCC	59.8	AGCCTGGCTTTGTTTAGCCT	60.4	157	
**Ac0862**		**(CGAT)6**	**CAGCTGTTCAGCAGTGAAGC**	**59.9**	**ACAGCTGCTCAAACGTCCTT**	**60.1**	**150**	**B/G/H/L/M/S**
			**GTGTCCGTCAACTCGTTCCT**	**60.2**	**ACAGCTGCTCAAACGTCCTT**	**60.1**	**214**	
**Ac3924**		**(TTGT)5**	**CGTCATTGTATAAACCACGCA**	**59.5**	**TGGTTATTTCCGCCTTTCTG**	**60.1**	**250**	**G/L**
Ac3974		(TTTG)7	TCGAAGTCTGGATTTGACCC	60.1	TCGTTTCCACTCTGAACTGTTG	60.3	159	H
			ACATGGAGGACGGAGACATC	59.9	TCGTTTCCACTCTGAACTGTTG	60.3	180	
**Ac3982**		**(GT)13**	**ACCACAGAAATGAACACGCA**	**60.2**	**TGAACATGGAGCTGAAGTGC**	**60.0**	**242**	**G/H/L/M**
			**ACCACAGAAATGAACACGCA**	**60.2**	**GATAAAGAGGCAAAAGCCCC**	**60.0**	**265**	
			**ACCACAGAAATGAACACGCA**	**60.2**	**ACACACTTAATGCAGCACGC**	**59.9**	**182**	
Ac4090		(AAAC)5	CTTCGTCTCTCAGCCTGGTC	60.1	AAGGCGATTCAAAGTGCTGT	59.9	164	L
**Ac4068**		**(AC)12**	**TGTCCCTGGACATACTTCACA**	**59.0**	**CCAAACGTTGTCATGCTCTG**	**60.3**	**202**	**G/H/L**

Locus	GB acc nr	Repeat motif	FORWARD PRIMER1 (5'-3')	Tm(ºC)	REVERSE PRIMER1 (5'-3')	Tm(ºC)	size (bp)	Tissue
Ac4114		(TGA)8	TTCGGGGAACTTTTGATACG	59.9	ATTCGAGCATGGTCATTTCC	59.9	192	B/G/L/S
			ATTCATCCCACTGAAGGGGT	60.6	ATTCGAGCATGGTCATTTCC	59.9	121	
**Ac4134**		**(ATT)10**	**CGTTCCCTTTCACCTCTCAA**	**60.2**	**CCCTGCTGTAAGCAAAACAA**	**59.0**	**243**	**G/H/M/S**
			**CGTTCCCTTTCACCTCTCAA**	**60.2**	**TGAATAATTCCAACCCTGCTG**	**59.9**	**256**	
**Ac4132**		**(TTG)8**	**CTGCTTCCAGTTGCTTCACA**	**60.2**	**AAATGAGAGACAAACACGACCA**	**59.6**	**184**	**G/M**
Ac4288		(ATT)11	TCTCATAGGAATGAACGGGG	59.9	TTTATACGAGTTGCCCCAGG	60.0	252	G/L/M/S
			TCTCATAGGAATGAACGGGG	59.9	GTGCAGCCATTACAAAGCAA	59.9	222	
**Ac0519**		**(GT)54**	**AACCTGCAGAAAGCCAAAGA**	**60.0**	**CAGCCTCTCTCTCTCTCCGA**	**60.0**	**240**	**B/G/H/L/M/S**
Ac4246		(CA)12acacacgcacgcacg(CA)15	GAGCAGCAGGATCAAGGTTC	60.0	TGTGTGTGTGTCGTGTGTGTC	60.2	280	G/(L)/S
**Ac1194**		**(GAT)8**	**CGTCTCTTTCACAGCGTTTC**	**58.7**	**TGTGCAGCCCTTTAATCTGA**	**59.4**	**215**	**H/L**
Ac0518		(GT)72	CATTTTCCATCTGGACTGGC	60.5	CAGCCTCTCTCTCTCTCCGA	60.0	279	B/G/H/L/M/S
			CATTTTCCATCTGGACTGGC	60.5	ACTGAGAGAGAGCCACGGTC	59.6	226	
**Ac0520**		**(GT)53**	**AACCTGCAGAAAGCCAAAGA**	**60.0**	**TTGGAAAAACTCATCCAGCC**	**60.1**	**241**	**B/G/H/L/M/S**
			**AACCTGCAGAAAGCCAAAGA**	**60.0**	**ATCCAGCCTATTCAGGCAGA**	**59.8**	**229**	
Ac0521		(GT)71	CATTTTCCATCTGGACTGGC	60.5	TTGGAAAAACTCATCCAGCC	60.1	280	B/G/H/L/M/S
			CATTTTCCATCTGGACTGGC	60.5	ATCCAGCCTATTCAGGCAGA	59.8	268	
**Ac4975**		**(GAAT)5**	**GCCAATGTGCTGCCTAAACT**	**60.3**	**TTTCGAGAATGACCATGCAA**	**60.2**	**198**	**G/H**
			**GTGCAACTTATTGCTGCCCT**	**60.3**	**TTTCGAGAATGACCATGCAA**	**60.2**	**250**	
Ac5056		(ATGT)6	TTCATGACAAGGTCGCAAAT	59.1	ACAGCCCAACTTAGCTCTGC	59.6	251	B/H/M
			TTCATGACAAGGTCGCAAAT	59.1	TCTGCTTTGCTGTGACCACT	59.6	236	
**Ac5167**		**(AC)18**	**ATCGATGACTGTCCCTCGTC**	**60.1**	**TTTTCCATCCAGTCACCCAT**	**60.2**	**116**	**G/H/M**
			**GCAGTTCTACAAACTCGCCC**	**59.9**	**TTTTCCATCCAGTCACCCAT**	**60.2**	**273**	
			**TTTCATCAGCAGACGGACAC**	**59.8**	**TTTTCCATCCAGTCACCCAT**	**60.2**	**158**	
Ac5239		(TTTG)5	TTTGGATGTTTTTGCAAAGTT	57.4	AAATTGCATTGTCCATTGTGA	58.9	242	L
**Ac1193**		**(CAT)8**	**TGCTGCAGCTGAAGAAGAAA**	**60.0**	**TGACATGAAGCAGGAGCACT**	**59.6**	**248**	**G/L/S**
			**CACCCTGCTGTGTGGATATG**	**60.0**	**TGACATGAAGCAGGAGCACT**	**59.6**	**154**	
			**GTTTCATTGGGAAGAAGGCA**	**60.1**	**TGACATGAAGCAGGAGCACT**	**59.6**	**217**	
Ac5337		(TTA)8	GGACTGTAGCTCGTCTGCAA	59.2	GAGCCACGTCTGAAGCTTTT	59.6	106	G/H/L
			CAGCAGGTTGTACTGGATGG	59.2	GAGCCACGTCTGAAGCTTTT	59.6	124	
**Ac5362**		**(TCAT)5**	**GCGTTTAATCATCAGGACCAG**	**59.6**	**GCACTTGGTGTCAGTTGCAT**	**59.8**	**276**	**G/H**
			**TCCACCAATAAAACCTCAGGA**	**59.4**	**GGATTAACAGCCAAGCCAAA**	**60.1**	**262**	
Ac0871		(AC)15	CCCCCTTACTTCACACTCCA	60.0	TGATATTATGAGGGTCGGGG	59.6	137	B/G/L/M/S
			CCCCCTTACTTCACACTCCA	60.0	GAGGGTCGGGGAGTAAAGAT	59.4	128	
**Ac0863**		**(CGAT)6**	**CAGCTGTTCAGCAGTGAAGC**	**59.9**	**ACAGCTGCTCAAACGTCCTT**	**60.1**	**150**	**B/G/H/L/M/S**
			**GTGTCCGTCAACTCGTTCCT**	**60.2**	**ACAGCTGCTCAAACGTCCTT**	**60.1**	**214**	
Ac5610		(TTA)9	GGGGAAAGGAACAGTCGTTA	59.0	GGTCTAACTTTGAAGGTTTTGGG	60.2	155	H
			GGGGAAAGGAACAGTCGTTA	59.0	TTTGAAGGTTTTGGGTTTATTGA	59.8	147	
**Ac5786**		**(TG)13**	**CCCAGGACGACAGAGTGTTT**	**60.2**	**CCTCTGGTGTCGGACAGTTT**	**60.2**	**123**	**B/G/L/M**
			**AGGCAGACAGAGAGAGACCG**	**59.7**	**CCTCTGGTGTCGGACAGTTT**	**60.2**	**166**	

Locus	GB acc nr	Repeat motif	FORWARD PRIMER1 (5'-3')	Tm(ºC)	REVERSE PRIMER1 (5'-3')	Tm(ºC)	size (bp)	Tissue
Ac1227		(TCAA)5	CTCAGCATCCTCACACAGGA	60.0	TTGAAGGAACTGACGGGAAC	60.1	111	B/G/H/L/M/S
			ATCTTCTGCAGCCTCAGCAT	60.1	TTGAAGGAACTGACGGGAAC	60.1	123	
**Ac6224**		**(TTA)10**	**ATTCCCCTGATTTGTGATTTT**	**57.4**	**ACGTGCCATTCTCACATCAA**	**60.1**	**238**	**G**
			**TTCCCCTGATTTGTGATTTTA**	**57.1**	**GTTCAGTGTCACAACCACGG**	**60.0**	**169**	
Ac6218		(TAT)8	GGTGCACTGAGATTGACCCT	60.1	GGGACAACCCACTCTCAAAA	59.9	184	H
			GGTGCACTGAGATTGACCCT	60.1	GGCAATCAGTCCCACAGTCT	60.1	225	
**Ac6356**		**(ATT)8**	**CTCGGTTGCCTATTTGATCC**	**59.5**	**TCAAAGTGGTGCTGTGAAGC**	**60.0**	**157**	**G/H**
			**CTCGGTTGCCTATTTGATCC**	**59.5**	**AATGATGACCAAAACCGGAA**	**60.2**	**127**	
Ac6327		(CTT)9	TGCGAACTGTTGATTTCTGC	60.0	ATAACTCCAGATGATGGCGG	59.9	117	B/H/L/M/S
**Ac6528**		**(CAA)9**	**AAAAACTGTGCCAAGCACCT**	**59.8**	**TGATCATCATTGATTTGCAGC**	**59.6**	**242**	**H**
			**TGTGCCAAGCACCTACAAAA**	**60.3**	**TGATCATCATTGATTTGCAGC**	**59.6**	**236**	
Ac6556		(TAT)8	TCCAGCTGGGGATATAGGTG	59.9	AACGCTTAATACCATGGGACA	59.4	186	G/H
			TGCTACCTGTTGCTGGATTG	59.9	AACGCTTAATACCATGGGACA	59.4	264	
**Ac1245**		**(CCAG)6**	**AGACGAAAAGCAAGGCGATA**	**60.0**	**CGGCCTCACATCGTTTTATT**	**60.0**	**244**	**L/S**
			**AGCAAGGCGATAAGGACGTA**	**59.9**	**CGGCCTCACATCGTTTTATT**	**60.0**	**236**	
**Ac6660**		**(AAACA)6**	**TACAGGAATCAAAGCCGGAC**	**60.1**	**GATAACACGGAGCACGGAAT**	**60.0**	**247**	**L/M**
Ac6666		(CAT)8	CTCATGCAGGGACCTCTCTC	59.9	CATTTCCATCAGATTGCAGG	59.1	132	B/G/H/M
			AGTCTGTGCACTCCCCTCAC	60.3	CATTTCCATCAGATTGCAGG	59.1	204	
			GTACCAAACTGCCCACAACA	59.5	CATTTCCATCAGATTGCAGG	59.1	103	
**Ac1314**		**(TTGA)5**	**ATGAAAAGCTGACTTGCCGT**	**59.9**	**GTATCAGCGGGCATAACCAT**	**59.8**	**254**	**G/S**
Ac1391		(GATT)6	CGCCCCCTCTACATCTGATA	60.1	TCAGAGAGGAAAACAAAGAAACA	58.2	227	H
**Ac6912**		**(ATA)8**	**TGTGATCAGGTCTCTCGTGC**	**60.0**	**GCCTGTGAGCAGATGAATGA**	**60.0**	**200**	**G/H**
			**TCGTGCTGCAGAACTTCACT**	**59.8**	**GCCTGTGAGCAGATGAATGA**	**60.0**	**186**	
**Ac6936**		**(CA)12**	**CTGGTACAGCAGAACACACACA**	**59.9**	**TTCATACGTGAAGGTGACGC**	**59.7**	**192**	**G/H/L**
Ac1287		(AAG)9	ACTCCATACCAGTTGGTGGC	59.9	AGAAGCTACCAGGCTACCCG	60.8	119	G/M/S
**Ac7204**		**(AAT)11**	**GGGTTGTTGAGTCAGCAGGT**	**60.2**	**TGATATTGTACGCTTTGTTTGTTTT**	**59.1**	**249**	**G/L**
			**GCCTGAAGGTGACGATGTCT**	**60.3**	**TGATATTGTACGCTTTGTTTGTTTT**	**59.1**	**212**	
Ac1255		(TAT)9	GACACGCGAGATGTGTGTTT	59.8	TCAGCAGTTAAGTGAAACTACTCCC	60.2	125	H/M/S
			TGGGGTATTAAAATGCTGGG	59.7	TCAGCAGTTAAGTGAAACTACTCCC	60.2	106	
**Ac7251**		**(AC)12**	**TTCTTCCTGGGGTCAAACAC**	**59.9**	**TTCCCTTACTGAAGATAATTGTTGC**	**59.9**	**161**	**G/L/S**
			**CAGCGACTATTCTTCCTGGG**	**59.8**	**TTCCCTTACTGAAGATAATTGTTGC**	**59.9**	**170**	
Ac7419		(TTTG)5	AGGAGGGGGAGACTCACTGT	60.1	CAGTGCAGTAGCTAATGGGGA	60.3	113	H/M
			AATATGCCAGGGCCACATAA	60.2	CAGTGCAGTAGCTAATGGGGA	60.3	226	
			ATAAGAGGAATGGCCAGGGT	59.8	CAGTGCAGTAGCTAATGGGGA	60.3	210	
**Ac7911**		**(CA)14**	**CGGCTGTGACATCATTCAGT**	**59.7**	**CGCAGGACGGAGTTTTATTT**	**59.2**	**151**	**G/H/L/S**
			**AAGAAACTGATGACAGCGGC**	**60.4**	**CGCAGGACGGAGTTTTATTT**	**59.2**	**167**	
Ac8081		(ACAG)5	GTTTTGGCAAAGTTTGGAGG	59.6	CCTGGCACTTCCACATCCTA	61.1	118	B/G/L/S
			ACCCTGAAGAGTGTTTTGGC	59.2	CCTGGCACTTCCACATCCTA	61.1	130	

Tab. S4
